# Current landscape and future of dual anti-CTLA4 and PD-1/PD-L1 blockade immunotherapy in cancer; lessons learned from clinical trials with melanoma and non-small cell lung cancer (NSCLC)

**DOI:** 10.1186/s40425-018-0349-3

**Published:** 2018-05-16

**Authors:** Young Kwang Chae, Ayush Arya, Wade Iams, Marcelo R. Cruz, Sunandana Chandra, Jaehyuk Choi, Francis Giles

**Affiliations:** 1 Developmental Therapeutics Program of the Division of Hematology Oncology, Early Phase Clinical Trials Unit, 645 N. Michigan Avenue, Suite 1006, Chicago, IL 60611 USA; 2 Robert H. Lurie Comprehensive Cancer Center of Northwestern University, 645 N. Michigan Avenue, Suite 1006, Chicago, IL 60611 USA; 3 Northwestern University Feinberg School of Medicine, 645 N. Michigan Avenue, Suite 1006, Chicago, IL 60611 USA

## Abstract

Immunotherapy is among the most rapidly evolving treatment strategies in oncology. The therapeutic potential of immune-checkpoint inhibitors is exemplified by the recent hail of Food and Drug Administration (FDA) approvals for their use in various malignancies. Continued efforts to enhance outcomes with immunotherapy agents have led to the formulation of advanced treatment strategies. Recent evidence from pre-clinical studies evaluating immune-checkpoint inhibitors in various cancer cell-lines has suggested that combinatorial approaches may have superior survival outcomes compared to single-agent immunotherapy regimens. Preliminary trials assessing combination therapy with anti-PD-1/PD-L1 plus anti-CTLA-4 immune-checkpoint inhibitors have documented considerable advantages in survival indices over single-agent immunotherapy. The therapeutic potential of combinatorial approaches is highlighted by the recent FDA approval of nivolumab plus ipilimumab for patients with advanced melanoma. Presently, dual-immune checkpoint inhibition with anti-programmed death receptor-1/programmed cell death receptor- ligand-1 (anti-PD-1/PD-L1) plus anti-cytotoxic T lymphocyte associated antigen-4 (anti-CTLA-4) monoclonal antibodies (MoAbs) is being evaluated for a wide range of tumor histologies. Furthermore, several ongoing clinical trials are investigating combination checkpoint inhibition in association with traditional treatment modalities such as chemotherapy, surgery, and radiation. In this review, we summarize the current landscape of combination therapy with anti-PD-1/PD-L1 plus anti-CTLA-4 MoAbs for patients with melanoma and non-small cell lung cancer (NSCLC). We present a synopsis of the prospects for expanding the indications of dual immune-checkpoint inhibition therapy to a more diverse set of tumor histologies.

## Background

The regulation of immune responses through MoAbs is a ground-breaking therapeutic strategy in oncology. Based on substantial pre-clinical and clinical evidence, several immunotherapy agents have received approval by the FDA as standard of care treatment for various malignancies over the past two decades [1, 2]. However, with increasing experience in the use of immunotherapy agents in clinical settings, several limitations, such as treatment resistance and undesired immunogenicity, have been observed [3, 4]. Extensive efforts have been made to meet such challenges, and novel immune checkpoints are being tested and are expected to pave the way for the next generation of immunotherapy agents [5].

The fundamental goal in advancing anti-cancer immunotherapy is to improve clinical outcomes. The use of combination checkpoint inhibition is being applied to meet this goal. This approach intends to exploit the distinct mechanisms of immunomodulation of two MoAbs in a single treatment regimen. Recent evidence suggests that the combined use of an anti-CTLA-4 immune-checkpoint inhibitor with an anti-PD-1/PD-L1 MoAb may have complementary action, thus yielding a higher clinical efficacy than either agent individually [6, 7]. Comprehensive data on the efficacy of MoAb combination therapy in clinical settings is warranted in order to ascertain the true therapeutic value of this treatment strategy.

Presently, combination checkpoint inhibition is being extensively evaluated for potential clinical benefit in a large number of tumor histologies. Due to positive outcomes in preliminary trials, nivolumab (IgG4 anti-PD-1 MoAb) plus ipilimumab (fully humanized IgG1 anti-CTLA-4 MoAb) is one of the most enthusiastically investigated combined immunotherapy regimens, with over 100 clinical trials in various stages [8, 9]. Of note, nivolumab plus ipilimumab received approval for use in BRAF V600 wild-type metastatic/unresectable melanoma, making it the first combination checkpoint inhibition regimen to be approved by the FDA [9]. In addition, other PD-1/PD-L1 inhibitors plus CTLA-4 inhibitor combination checkpoint inhibition regimens that are presently in clinical trials include atezolizumab (anti-PD-L1 MoAb) plus ipilimumab, pembrolizumab (IgG4 anti-PD-1 MoAb) plus ipilimumab, and tremelimumab (IgG2 anti-CTLA-4 MoAb) plus durvalumab (Fc optimized anti-PD-L1 MoAb) [10]. The data published from these trials will be crucial to appraise the efficacy of combination immune checkpoint inhibitor regimens in varying clinical scenarios.

In this review, we describe the rationale for combined immunotherapy with PD-1/PD-L1 plus CTLA-4 checkpoint inhibitors. Building on what we have learned through studies of combination checkpoint inhibition in patients with melanoma and NSCLC, we shall also critically assess the current landscape and future prospects for the development of an ideal combination checkpoint inhibition regimen.

### Role of PD-1/PD-L1 and CTLA-4 in modulation of anti-tumor T-cell activity

**Fig. 1:**
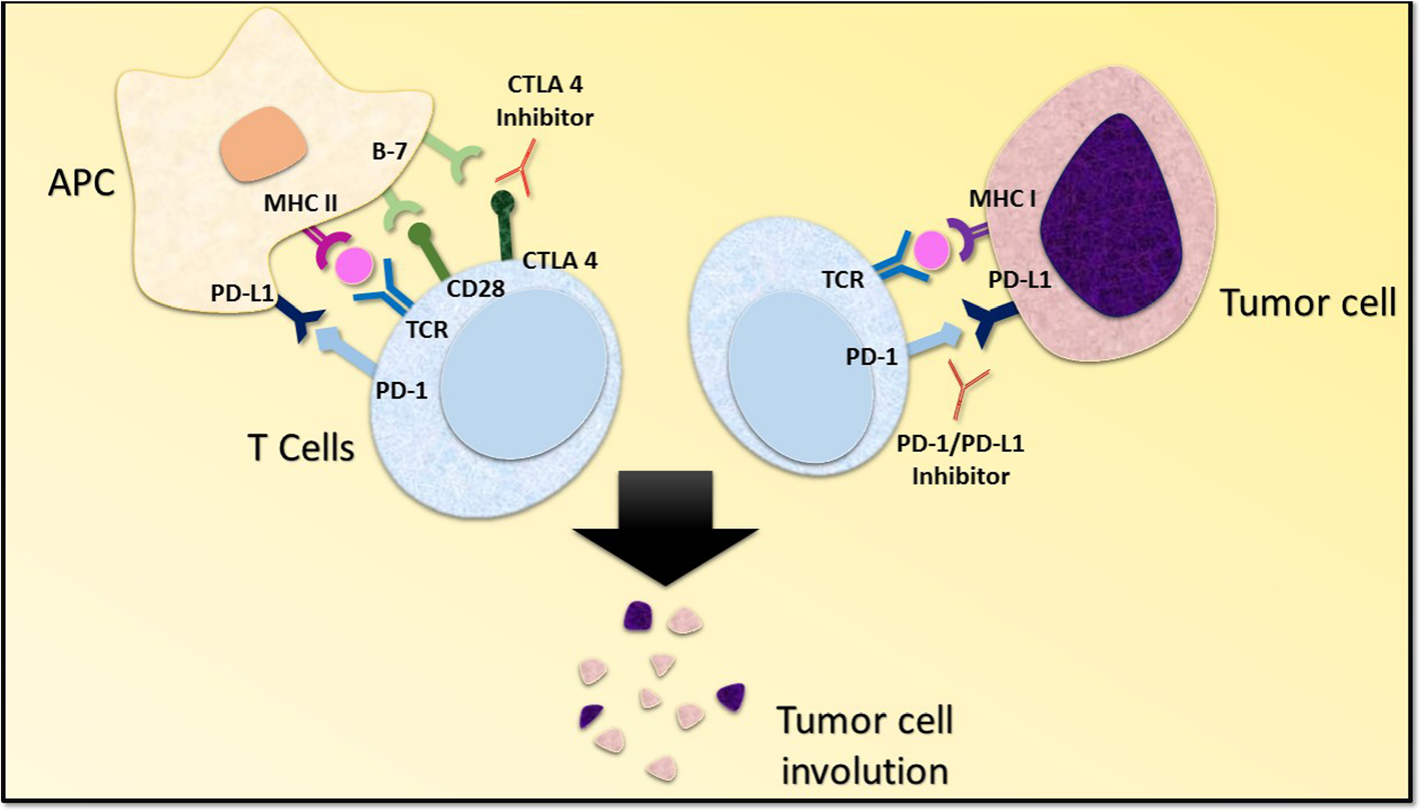
Mechanism of CTLA 4 and PD-1/PD-L1 inhibition. The activation of T cells is mediated by the interaction of T cell receptor and the CD28 receptor with class II major histocompatibility complex and B7 co-stimulatory molecule located on the antigen presenting cells. The interaction of CTLA-4 with the B7 molecule delivers an inhibitory signal, effectively checked by CTLA-4 inhibitors. On the other hand, the negative regulation of T cells resulting from PD-1/PD-L1 interaction between T cells and tumor cells is prevented by PD-1/PD-L1 inhibitors. Abbreviations: APC, antigen presenting cell; PD-1, programmed death receptor-1; PD-L1, programmed cell death receptor ligand-1; TCR, T cell receptor; MHC I, major histocompatibility complex class I; MHC II, major histocompatibility complex class II

The process of T cell activation requires two signals. The primary signal comes from the binding of the T cell receptor (TCR) to the major histocompatibility complex (MHC) molecule presented by an antigen presenting cell (APC) [11]. The costimulatory signal may arise from one of several distinct T cell-APC interactions. One such pathway is the engagement of CD28 on T cells with CD80 (B7–1) or CD86 (B7–2) on APCs [11] (Fig. 1). T-cell activity can be modulated by regulating the generation of costimulatory signals through various mechanisms.

Several signaling pathways have been implicated in the modulation of T cell activity. The CTLA-4 molecule is a homolog of CD28 and is expressed by T cells (Fig. 1) [12, 13]. The influence of CTLA-4 on T cell activity primarily occurs in the priming phase of T cell activation [14]. CTLA-4 competitively binds to B7 on APCs and inhibits the costimulatory signal that arises from the engagement of CD28 with B7, thereby diminishing the T cell immune response [13, 15–18]. The upregulation of CTLA-4 expression on CD8+ and CD4+ T cells precludes stimulatory signaling from CD28-B7 binding and TCR-MHC binding [11, 19]. On the other hand, regulatory T cells (T_reg_ cells) exhibit constitutive expression of CTLA-4 [20]. Murine models bearing T_reg_ cells deficient in *CTLA-4* exhibited attenuated immunosuppressive activity, thus highlighting the significance of CTLA-4 in regulating immunological self-tolerance [20].

The PD-1 molecule is akin to CTLA-4. It is a member of the B7-CD28 family and is expressed by myeloid derived cells, B cells, and T cells [21, 22]. PD-1 has two corresponding ligands, PD-L1 and PD-L2 [14]. PD-L1 is expressed by a diverse set of cells including hematopoietic cells, leucocytes, parenchymal cells, and tumor cells, whereas PD-L2 is expressed by dendritic cells and macrophages [22–24]. The PD-1 receptor on T cells binds PD-L1 expressed by APCs and inhibits pro-inflammatory events such as T cell proliferation and cytokine production (Fig. 1) [14, 25]. Of note, recent evidence has suggested that PD-1/PD-L1 interactions facilitate immune escape by tumor cells [24, 26]. This phenomenon has been attributed to PD-1/PD-L1 mediated induction of anergy and apoptosis of activated T cells, tumor resistance to the cytotoxic T cell response, and differentiation of CD4+ T cells into Fox3p + CD4+ T_reg_ cells [27–29].

An intricate knowledge of various pathways regulating T cell-APC interactions has been central to identifying the points of intervention that allow us to modulate host immune responses. The aforementioned evidence and other studies with similar outcomes prompted the development of PD-1/PD-L1 and CTLA-4 checkpoint inhibitors for potential application in anti-cancer therapy.

### Preclinical rationale for combination checkpoint inhibition

Combination immunotherapy with PD-1/PD-L1 plus CTLA-4 checkpoint inhibitors has been studied in multiple cancer cell lines. In an experiment on murine preclinical models, vaccination with B16-Flt-3 ligand (Fvax) along with the use of CTLA-4 antibody promoted tumor rejection in 10% of mice with pre-implanted B16-BL6 melanoma [7]. Fvax plus PD-1 blockade exhibited tumor rejection in 25% of mice. The combined use of CTLA-4 and PD-1 checkpoint inhibitors resulted in the rejection of B16-BL6 melanoma in 50% of test animals. Upon the addition of a PD-L1 inhibitor to the above, 65% of test animals exhibited rejection of melanoma. The outcomes observed with combined PD-1 and CTLA-4 blockade were found to correlate with an increase in CD4+ effector T cell (CD4+ T_eff_) to T_reg_ cell ratio and CD8+ T cell to T_reg_ cell ratio in tumor tissue. Another significant observation was that a high percentage of T cells positive for CTLA-4 and PD-1 that would have undergone anergy remained active with combined PD-1 plus CTLA-4 blockade [7].

Similar findings were documented in another study investigating the effects of checkpoint inhibition (PD-1/PD-L1 and CTLA-4) in murine models of ovarian (ID8-VEGF) and colonic carcinoma (CT26) [30]. For ID8-VEGF, tumor regression was observed in 25% of test animals after PD-1 blockade, 25% with CTLA-4 blockade, and 37.5% with PD-L1 blockade, as compared to 50% with combined CTLA-4 plus PD-1 or PD-L1 blockade. Likewise, in mice bearing CT26, tumor regression was observed in 25% of test animals with PD-1 blockade, 50% with CTLA-4 blockade, and 33% with PD-L1 blockade, as compared to 75% (*p* < 0.01 for the combination to checkpoint inhibitor monotherapy comparison) with combined CTLA-4 plus PD-1 or PD-L1 blockade. Combined therapy with CTLA-4 and PD-1 blockade was associated with a greater enhancement in tumor induced lymphocyte (TIL) activity and proliferation similar to CTLA-4 or PD-1 blockade alone [30]. Combination therapy also decreased the frequency of T_reg_ cells and functional markers of activated T_reg_ cells such as glucocorticoid induced tumor necrosis factor receptor (GITR) [30]. This suggested that dual checkpoint inhibition simultaneously blunted the function and decreased the number of T_reg_ cells.

In view of these and other preclinical studies, it was established that combined immune checkpoint blockade decreases suppression of the host immune system, while promoting inflammation in the tumor microenvironment. Moreover, the vast amount of preclinical data also suggested that the anti-tumor activity of combination therapy with CTLA-4 plus PD-1/PD-L1 checkpoint inhibitors may have superior outcomes compared to CTLA-4 or PD-1 monotherapy. It was thus prudent to investigate the therapeutic potential of CTLA-4 plus PD-1/PD-L1 checkpoint inhibitors in clinical settings.

### Emergence of combination checkpoint inhibition

Cancer immunosurveillance has been considered one of the primary natural mechanisms of defense against aberrant cell populations [31, 32]. Immune cells recognize and eliminate transformed cells through various cellular interactions [33]. In addition, the immune system is also involved in shaping tumor immunogenicity, a phenomenon theorized as immunoediting [34]. However, it was observed that cancer cells gradually undergo immune selection that disrupts the equilibrium with immune cells, consequently generating a tumor cell population that effectively evades immune surveillance [35–38]. Furthermore, alterations in the tumor microenvironment have also been reported to mediate immune escape [39]. It was thus theorized that therapeutic agents with the ability to restore immune surveillance or prevent immune escape of tumor cell populations could potentially have a significant impact in clinical oncology.

The knowledge of various factors that influence tumorigenesis has been exploited for developing a wide range of immunotherapy agents. Immunogenic cell death inducers, immunostimulatory cytokines, pattern recognition receptor agonists (PRR agonists), and tumor targeting antibodies have benefitted cancer patients for nearly two decades [40]. Numerous immunotherapy agents have received FDA approval for use as monotherapy in a variety of tumor histologies. For instance, nivolumab has been approved for patients with melanoma, renal cell carcinoma (RCC), metastatic squamous cell carcinoma of the head and neck (SCCHN), classical Hodgkin’s lymphoma (HL) and advanced lung cancer [41–46]. Pembrolizumab, another anti-PD-1 MoAb, is approved for use in patients with SCCHN, NSCLC, and melanoma [47–49]. Likewise, atezolizumab is approved for patients with NSCLC and urothelial carcinoma [50–52]. However, any further enhancements in clinical benefit beyond checkpoint inhibitor monotherapy are still in clinical trials.

Recent clinical trial data comparing combination therapy with nivolumab and ipilimumab versus ipilimumab monotherapy in treatment naïve melanoma patients drew much attention (CheckMate 069, NCT01927419). In patients with *BRAF*-wild type melanoma, the investigators reported an objective response of 61% (95% CI: 49–72) as opposed to 11% (95% CI: 3–25) with combination checkpoint inhibition and ipilimumab monotherapy, respectively [53]. Furthermore, 22% of study participants receiving combination therapy exhibited a complete response, as compared to none among those receiving ipilimumab monotherapy [53]. In view of these findings, combined therapy with nivolumab and ipilimumab in melanoma patients became the first FDA approved (accelerated approval) indication for combination checkpoint inhibition. Furthermore, this study prompted comprehensive efforts to explore the application of combined immunotherapy with anti-PD-1/PD-L1 and anti-CTLA-4 MoAbs for patients with various malignancies.

Currently there are a large number of clinical trials evaluating several PD-1/PD-L1 plus CTLA-4 checkpoint inhibitor combination regimens. Positive outcomes in preliminary trials have made way for more intensive efforts to explore the full potential of combination checkpoint inhibition regimens, particularly nivolumab plus ipilimumab. It is widely anticipated that the ongoing trials will provide formidable evidence to support clinical applications of PD-1/PD-L1 plus CTLA-4 checkpoint inhibitor combination regimens for patients with a substantial variety of tumor histologies.

## Methods

The details of pertinent clinical trials were gathered from clinicaltrials.gov (United States National Institute of Health) from the database available to public domain. The database was narrowed using the search queries “nivolumab” with “ipilimumab”, “pembrolizumab” with “ipilimumab”, “atezolizumab” with “ipilimumab” and “tremelimumab” with “durvalumab”. All prospective clinical trials using aforementioned immunotherapy agents as treatment intervention in NSCLC or melanoma patients were selected from the filtered results. Additionally, all phase 1 solid tumor trials aimed at providing a recommendation for the appropriate phase 2 dose of above combinations were also selected. The NCT number of each trial was used to search for published results on online databases including PubMed, American Association of Cancer Research (AACR), American Society of Clinical Oncology (ASCO) and European Society for Medical Oncology (ESMO).

### Clinical trials: Current landscape

**Fig. 2:**
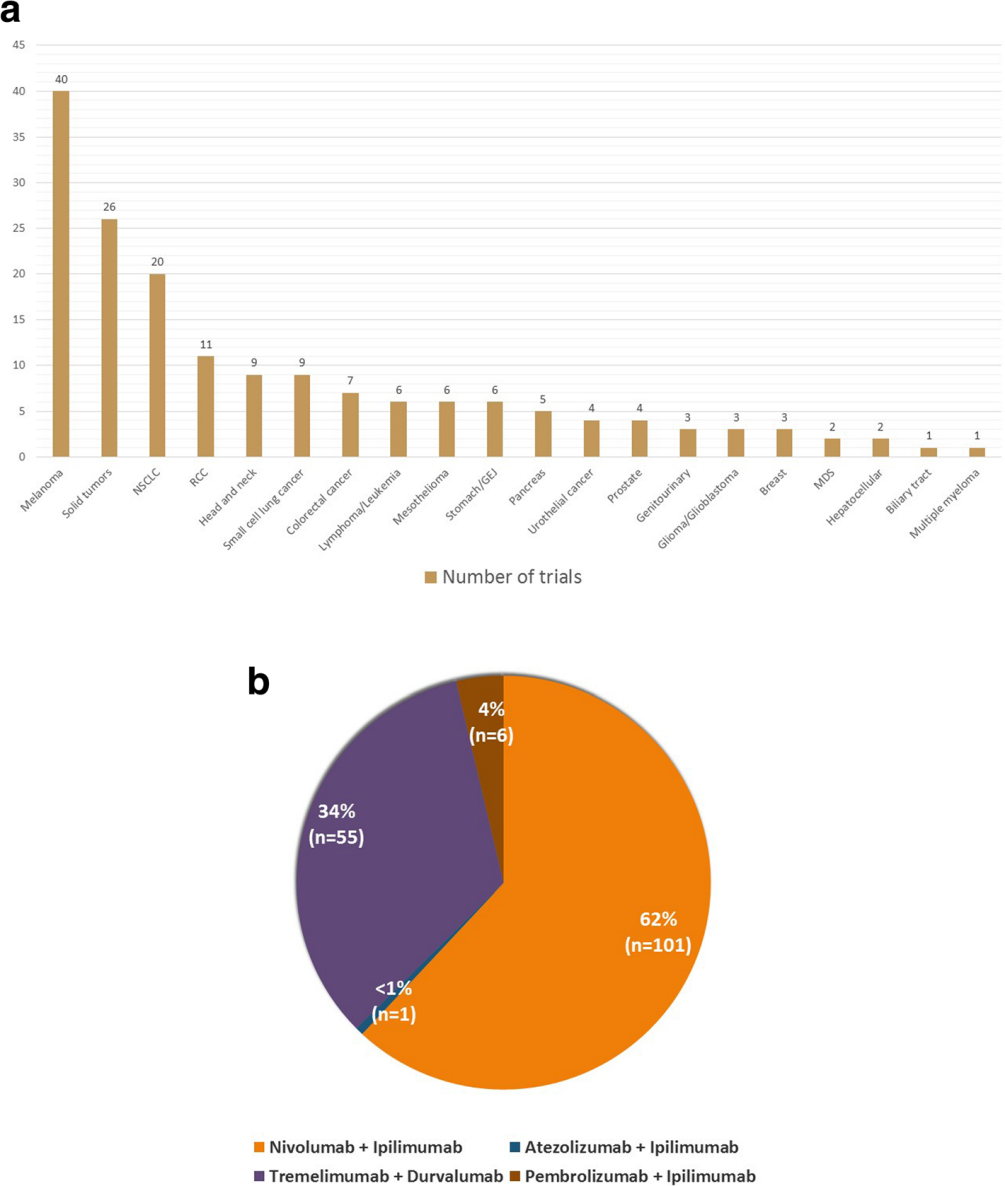
Current landscape of combination immunotherapy trials for various tumor histologies. **a** Number of combination checkpoint inhibition trials for various tumor histologies. Abbreviations: NSCLC, non-small cell lung carcinoma; RCC, renal cell carcinoma; GEJ, gastro-esophageal junction; MDS, myelodysplastic syndrome. **b** Landscape of combination checkpoint inhibition agents. Legend: The figure elaborates relative number of trials for four combinations of immunotherapy agents. Nivolumab plus ipilimumab: 62% (101 trials), pembrolizumab plus ipilimumab 4% (6 trials), tremelimumab plus durvalumab 34% (55 trials) and atezolizumab plus ipilimumab < 1% (1 trial)

The United States National Institutes of Health lists a total of 44 ongoing clinical trials (clinicaltrials.gov) evaluating combined immunotherapy with anti-PD-1/PD-L1 plus anti-CTLA-4 MoAbs for patients with NSCLC and melanoma (Tables 1 and 2, Fig. 2a). The designated combination therapy regimens include nivolumab plus ipilimumab, pembrolizumab plus ipilimumab, atezolizumab plus ipilimumab, and tremelimumab plus durvalumab. A majority of these trials focus on survival and other treatment response indices, whereas a limited number of trials are investigating the safety profile and maximum tolerable dose (MTD) of combination therapy protocols. Furthermore, eight phase 1/phase 2 clinical trials are in the process of enrolling participants with solid tumors in order to determine recommended phase 2 doses (RP2D) for combination checkpoint inhibitor therapy with various anti-PD-1/PD-L1 and anti-CTLA-4 MoAbs. Of note, over three quarters (37 of 44 trials) of ongoing combination therapy trials for patients with NSCLC and melanoma are investigating treatment regimens that involve the application of nivolumab and ipilimumab (Fig. 2a and b).

**Table 1:** Melanoma trials evaluating combination checkpoint inhibition with anti-CTLA-4 plus anti- PD-1/PD-L1 monoclonal antibodies

Cancer type	Phase	Primary outcome	Dosing regimen	Enrollment number	Status	Results	Clinical trials identification number
Unresectable Stage III/stage IV malignant melanoma	Phase 1	Assess the safety of specified doses of NIVO + IPI combination therapy for up to 5.5 years; secondary outcome: assessment of tumor response	Cohort 1: induction with NIVO 0.3 mg/kg (Q3W for 21 weeks) + IPI 3 mg/kg (Q3W for 9 weeks), maintenance with NIVO 0.3 mg/kg + IPI 3 mg/kg (Q12W for a total of 84 weeks); cohort 2: induction with NIVO 1 mg/kg (Q3W for 21 weeks) + IPI 3 mg/kg (Q3W for 9 weeks), maintenance with NIVO 1 mg/kg + IPI 3 mg/kg (Q12W for 84 weeks); cohort 3: induction with NIVO 3 mg/kg (Q3W for 21 weeks) + IPI 3 mg/kg (Q3W for 9 weeks), maintenance with NIVO 3 mg/kg + IPI 3 mg/kg (Q12W for 84 weeks); cohort 4: induction with NIVO 10 mg/kg (Q3W for 21 weeks) + IPI 3 mg/kg (Q3W for 9 weeks), maintenance with NIVO 10 mg/kg + IPI 3 mg/kg (Q12W for a total of 84 weeks); cohort 5: induction with NIVO 10 mg/kg (Q3W for 21 weeks) + IPI 10 mg/kg (Q3W for 9 weeks), maintenance with NIVO 10 mg/kg + IPI 10 mg/kg (Q12W for 84 weeks); cohort 6: NIVO 1 mg/kg (Q2W for 96 weeks); cohort 7: NIVO 3 mg/kg (Q2W for 96 weeks); cohort 8: 4 doses of IPI 3 mg/kg + NIVO 1 mg/kg (Q3W for 12 weeks), then monotherapy with NIVO 3 mg/kg (Q2W for 96 weeks)	136	Completed, results available	53% patients had grade 3–4 AE; NIVO 0.3 mg/kg + IPI 3 mg/kg (*n* = 14): 1 year OS rate = 56%, median OS = 14.8 months; NIVO 1 mg/kg + IPI 3 mg/kg (*n* = 17): 1 year OS rate = 94%, median OS non recordable; NIVO 3 mg/kg + IPI 1 mg/kg (*n* = 16): 1 year OS rate = 89%, median OS non recordable; NIVO 3 mg/kg + IPI 3 mg/kg (*n* = 6): 1 year OS rate = 100%, median OS non recordable; concurrent NIVO + IPI (*n* = 53): 1 year OS rate = 82%, median OS = 39.7 months; sequenced regimen (NIVO + IPI followed by monotherapy with NIVO Q2W for 48 doses, *n* = 32): median OS = 13.0 months, insufficient follow-up of 1 year OS rate. Subgroup analysis: OR in PD-L1 positive tumors: concurrent therapy: 6 of 13 patients, sequential therapy: 4 of 8 patients; OR in PD-L1 negative tumors: concurrent therapy: 9 of 22 patients; sequential therapy: 1 of 13 patients	NCT01024231 [54]
Advanced melanoma	Phase 1	Incidence of serious AE and treatment emergent AE; secondary outcomes: PFS, OS, ORR and duration of response (assessed up to week 49)	Arm 1: NIVO Q2W + TAK 580 QW; arm 2: plozalizumab 2 mg QW for weeks 1, 3, 5 and 9, then plozalizumab Q4W + NIVO Q2W; arm 3: IPI (once in week 3, 6, 9 and 12) + NIVO (administered in weeks 3, 6, 9, 12 and 15, Q2W thereafter) and vedolizumab (once in weeks 1, 3, 5 and 13)	156	Recruiting	NA	NCT02723006
Stage III unresectable or stage IV melanoma	Phase 1b	To determine MTD of specified treatment regimen (time frame: 12 months); secondary outcome: ORR	NIVO + IPI and ACY 241	36	Recruiting	NA	NCT02935790
BRAF mutant metastatic/unresectable melanoma	Phase 1 RCT	Incidence of AE (≥ grade 3 NCI CTCAE v4.0) evaluated up to 3 weeks after induction therapy with IPI; secondary outcomes: disease control rate and RR evaluated till 4 weeks after completion of therapy; proportion of patients with ≥ grade 3 AE after progression of disease on IPI	Arm A1: trametinib QID + dabrafenib BID for the first 25 days, then 4 courses of IPI Q3W; arm A2: 25 days of trametinib QID + dabrafenib BID, then 4 courses of IPI + NIVO Q3W, maintenance with NIVO Q2W (42 courses); arm B1: 25 days of trametinib QID, then 4 courses of IPI Q3W; arm B2: 25 days of trametinib QID, then 4 courses of IPI + NIVO Q3W, maintenance with NIVO Q2W (42 courses); arm C1: dabrafenib BID for 25 days, then 4 courses of IPI Q3W; arm C2: 25 days of dabrafenib BID, then 4 courses of IPI + NIVO Q3W, maintenance with NIVO Q2W (42 courses); arm D1: 4 courses of IPI Q3W; arm D2: 4 courses of IPI + NIVO Q3W, maintenance with NIVO Q2W (42 courses)	40	Recruiting	NA	NCT01940809
Stage IIIC/IV skin melanoma	Phase 1/Phase 2	Assess the safety of adjuvant NIVO + low-dose IPI	Low fixed dose NIVO in combination with low fixed dose IPI	6	Recruiting	NA	NCT02941744
Uveal melanoma with liver metastases	Phase 1/Phase 2	Evaluate tolerance and safety profile for treatment regimen (time frame: 3 years); secondary outcomes: PFS and RR (as per RECIST criteria)	Yttrium 90 followed by 4 doses of NIVO 1 mg/kg + IPI 3 mg/kg Q3W; maintenance with NIVO 3 mg/kg monotherapy Q2W for a maximum of 3 years	18	Not yet recruiting	NA	NCT02913417
Metastatic melanoma	Phase 1/ Phase 2 RCT	Evaluation of grade 3–4 toxicity (treatment associated) event-free survival for up to 6 months	Active comparator: 4 doses of NIVO 1 mg/kg IV + IPI 3 mg/kg IV Q3W, then monotherapy with NIVO 3 mg/kg Q2W; experimental arm: 4 doses of NIVO 1 mg/kg IV + IPI 0.3 mg/kg IT Q3W, followed by monotherapy with NIVO 3 mg/kg Q2W	65	Recruiting	NA	NCT02857569 (NIVIPIT)
Recurrent/advanced melanoma	Phase 1/Phase 2 RCT	Pathological complete RR; secondary outcome: ORR, PFS (time frame: 5 years)	Arm A: 3 doses of NIVO 3 mg/kg Q2W, then surgery at weeks 6 to 8; maintenance with NIVO 3 mg/kg Q3W after surgery; Arm B: 2 doses of IPI 3 mg/kg + NIVO 1 mg/kg Q3W and then surgery; maintenance with 2 doses of NIVO 1 mg/kg + IPI 3 mg/kg Q3W, continued as NIVO 3 mg/kg at Q3W	66	Recruiting	NA	NCT02736123
Advanced melanoma/renal cell carcinoma	Phase 1/ Phase 2 RCT	PFS, the number of study participants with AE (evaluation for up to 2 years), those discontinuing of study due to AE (evaluation for up to 2 years) and the patients that experienced DLT (assessed up to 6 weeks); secondary outcomes: OS, duration of response and ORR for phase 1b and 2	Arm 1: PEMBRO monotherapy; arm 2: two 6-weeks cycles of PEMBRO + IPI Q3W; arm 3: pegIFN-2b QW for each cycle (cycle duration: 6 weeks) + PEMBRO Q3W	343	Completed	(October 17, 2016 cut-off date): 153 participants received a minimum of 1 dose of PEMBRO + IPI; 72% (110 of 153 patients) received all 4 doses of PMEBRO + IPI; 42% (64 of 153 patients) were on PEMBRO monotherapy; - TRAEs: 45% (69 of 153 patients) developed grade 3–4 TRAEs, 51% (78 of 153 patients) developed grade 1–2 TRAEs; irAEs: seen in 60% (92 of 153) participants, 27% (42 of 153 patients) documented to have grade 3–4 irAEs OR: 61% (93 of 153 patients, 95%CI: 53–69) - CR: 15% (23 of 153 patients) - PR: 46% (70 of 153 patients) - SD: 18% (28 of 153 patients) - PD: 19% (29 of 153 patients) - PFS at 12 months: 69% (95%CI: 60–75)	NCT02089685; KEYNOTE-029 [58]
Melanoma with leptomeningeal metastases	Phase 2	OS rate assessed at 2 years	Pre-determined doses of IPI + NIVO combination therapy, then monotherapy with NIVO, duration of each treatment cycle to be 6 weeks	18	Not yet recruiting	NA	NCT02939300
Resected stage IIIB/IIIC/IV melanoma	Phase 2	Evaluation of adverse effects with specified treatment regimen; secondary outcomes: time to relapse and immunological response	Cycle 1: 4 doses of IPI 1 mg/kg + NIVO 3 mg/kg Q3W for 12 weeks; cycles 2, 3, 4 and 5: monotherapy with NIVO 480 mg Q4W for 48 weeks	25	Not yet recruiting	NA	NCT02970981
Stage III/resected stage IV melanoma	Phase 2	Recurrence free survival and OS; evaluate toxicity of adjuvant low-dose IPI and NIVO for up to 7 months	Adjuvant therapy with NIVO 3 mg/kg Q2W + IPI 1 mg/kg Q6W, for a total of 6 months duration	25	Recruiting	NA	NCT02656706; BrUOG 324
Uveal melanoma	Phase 2	OS at 12 months	4 doses of IPI + NIVO Q3W, then NIVO monotherapy Q2W	48	Recruiting	NA	NCT02626962; GEM1402
Uveal melanoma	Phase 2	ORR at 12 weeks	Induction phase: 4 doses of IPI 3 mg/kg + NIVO 1 mg/kg at week 1, 4, 7 and 10, continued through week 12; maintenance phase: monotherapy with NIVO 3 mg/kg Q2W for study participants with unmanageable toxicity or no progression after 12 weeks of induction therapy, to be continued till progression or unacceptable toxicity	52	Recruiting	NA	NCT01585194
Advanced melanoma	Phase 2	RR, evaluated up to 16 weeks; secondary outcomes: PFS assessed up to 24 months and safety	IPI + PEMBRO (all study participants to have received initial PD-1/PD-L1 antibody therapy prior to enrollment as per selection criteria)	70	Recruiting	NA	NCT02743819
Advanced mucosal/acral lentiginous melanoma	Phase 2	OR rate for mucosal melanoma (assessed up to 2 years); secondary outcomes: OR rate for acral lentiginous melanoma, PFS, OS	Induction phase: 4 doses of IPI 3 mg/kg + NIVO 1 mg/kg Q3W; maintenance phase: monotherapy with NIVO 3 mg/kg Q2W for 48 doses; NIVO monotherapy to be continued for another 12 weeks in study participants demonstrating CR	72	Not yet recruiting	NA	NCT02978443
Melanoma	Phase 2	Evaluation of clinical benefit rate up to 6 months	Induction: IPI + NIVO; maintenance phase: NIVO only	110	Recruiting	NA	NCT02320058; CheckMate 204
Advanced melanoma/bladder cancer	Phase 2	Evaluation of RR up to 12 weeks	Treatment regimen for melanoma patients: 4 doses of NIVO 1 mg/kg + IPI 3 mg/kg Q2W	120	Recruiting	NA	NCT02553642
Stage III/IV melanoma with progression/relapse on PD-1 inhibitor therapy	Phase 2 RCT	Pathological CR with neoadjuvant IPI + NIVO combination therapy and neoadjuvant NIVO monotherapy, assessed at day 57	Group A: Neoadjuvant therapy with NIVO 3 mg/kg on weeks 1, 3, 5 and 7, then surgical excision, later adjuvant phase with NIVO 3 mg/kg Q2W for 6 months; group B: neoadjuvant therapy with IPI 3 mg/kg and NIVO 1 mg/kg on weeks 1, 4 and 7, then surgical excision and later adjuvant therapy same as group A	40	Recruiting	NA	NCT02519322
Stage III/IV melanoma with progression/relapse on PD-1 inhibitor therapy	Phase 2 RCT	OR as per RECIST v1.1 (time frame: 18 weeks)	Experimental arm 1: 4 doses of NIVO 1 mg/kg + IPI 3 mg/kg Q3W; experimental arm 2: 4 doses of IPI 3 mg/kg monotherapy Q3W	70	Recruiting	NA	NCT02731729
Melanoma with brain metastases	Phase 2 RCT	Intracranial response rate assessed for up to 3 years; secondary outcomes: ORR, PFS, OS, extracranial response	Cohort 1: monotherapy with NIVO 3 mg/kg Q2W; cohort 2: monotherapy with NIVO 3 mg/kg Q2W; cohort 3: 4 doses of NIVO 1 mg/kg + IPI 3 mg/kg Q3W, followed by NIVO 3 mg/kg Q2W	76	Recruiting	NA	NCT02374242
Stage III melanoma	Phase 2 RCT	Pathological response rate at 6 weeks; RR (assessed up to 6 weeks); incidence of treatment related AE (time frame: 12 weeks)	Arm A: 2 cycles of NIVO 1 mg/kg + IPI 3 mg/kg before surgery at week 6; arm B: 2 cycles of NIVO 3 mg/kg + IPI 1 mg/kg before surgery at week 6; arm C: 2 cycles of NIVO 3 mg/kg + IPI 3 mg/kg before surgery at week 6	90	Recruiting	NA	NCT02977052
Advanced/metastatic melanoma	Phase 2 RCT	Determining the percent candidates that develop grade 3–5 AE attributable to induction therapy (time frame: 25 weeks); secondary outcomes: investigator assessed duration of response, RR and rate of progression	Cohort A: NIVO preceding IPI: induction with 6 doses of NIVO 3 mg/kg IV Q2W, to be continued in maintenance phase for up to 2 years; 4 doses of IPI 3 mg/kg Q3W in induction phase only; Cohort B: IPI preceding NIVO: 4 doses of IPI 3 mg/kg Q3W in induction phase only; 6 doses of NIVO 3 mg/kg IV Q2W during induction, continued in maintenance phase for up to 2 years	177	Results available	- Grade 3–5 TRAEs Cohort A: 34 of 68 patients (95%CI: 37.6–62.4); Cohort B: 30 of 70 patients (95%CI: 31.1–55.3) - Response at 25 weeks: Cohort A: 41% (28 patients, 95%CI: 29.4–53.8); Cohort B: 20% (14 patients, 95%CI: 11.4–31.3) - 12 months OS: Cohort A: 76% (95%CI: 64–85) Cohort B: 54% (95%CI: 42–65)	NCT01783938; CheckMate 064 [57]
Previously untreated unresectable/metastatic melanoma	Phase 2 RCT	Percent study participants (BRAF wild-type) with OR (time frame: 6 months or more)	Experimental regimen 1: 4 doses of NIVO 1 mg/kg + IPI 3 mg/kg Q3W followed by monotherapy with NIVO 3 mg/kg Q2W; experimental regimen 2: placebo + 4 doses of IPI 3 mg/kg Q3W followed by placebo Q2W	179	Completed, results available	Participants with BRAF wild-type tumors: OR and CR with placebo: 11% (4 of 37 patients, 95%CI: 3 to 25) and 0%, respectively; OR and CR with combination therapy: 61% (44 of 72 patients, 95%CI: 49 to 72) and 22% (16 patients), respectively; HR = 0.40 (95%CI: 0.23 to 0.68, p < 0.001) for death or progression of disease when comparing IPI + NIVO combination therapy versus IPI + placebo. Participants with BRAF mutant tumors: OR and CR with placebo: 10% (1 of 10 patients, 95%CI: 0 to 45) and 0%, respectively; OR and CR with combination therapy: 52% (12 of 23 patients, 95%CI: 31 to 73) and 22% (5 patients), respectively; HR = 0.38 (95%CI: 0.15 to 1.00); Subgroup analysis: OR rate with combination therapy: PD-L1 positive tumors: 58, 95%CI: 37 to 78, PD-L1 negative tumors: 55, 95%CI: 41 to 69); OR with IPI monotherapy: PD-L1 positive tumors: 18% (95%CI: 2 to 52), PD-L1 negative tumors: 4% (95%CI: 0 to 19)	NCT01927419; CheckMate 069 [53]
Melanoma	Phase 2 RCT	Evaluation of the best ORR at 18 weeks; secondary outcomes: OS and PFS	Experimental arm: induction with cobimetinib + vemurafenib for 6 weeks followed by NIVO + IPI; Control arm: NIVO + IPI only	200	Not yet recruiting	NA	NCT02968303
BRAF mutant metastatic melanoma	Phase 2 RCT	OS evaluated up to 24 months; secondary outcomes: PFS (time frame: 2 years), 3 year PFS rate, duration of response (evaluated for 24 months)	Arm A: binimetinib 45 mg BID + encorafenib 450 mg OD till progression, then 4 doses of NIVO 1 mg/kg + IPI 3 mg/kg Q3W and later NIVO 3 mg/kg Q2W; arm B: 4 doses of IPI 3 mg/kg + NIVO 1 mg/kg Q3W, later NIVO 3 mg/kg Q2W till disease progression, binimetinib 45 mg BID + encorafenib 450 mg OD till progression; arm C: binimetinib 45 mg BID + encorafenib 450 mg OD for 8 weeks, then 4 doses of NIVO 1 mg/kg + IPI 3 mg/kg Q3W and later NIVO 3 mg/kg Q2W till progression. Thereafter, treatment continued with binimetinib 45 mg BID + encorafenib 450 mg OD till progression of disease	230	Recruiting	NA	NCT02631447; SECOMBIT study
Malignant melanoma	Phase 2 RCT	Evaluation of efficacy with adjuvant therapy with NIVO + IPI or NIVO monotherapy (time frame: 24 months)	Active comparator: Placebo + NIVO: IPI substituted with placebo for weeks 1, 4, 7 and 10; NIVO substituted with placebo for weeks 4 and 10; Experimental regimen: 4 doses of IPI 3 mg/kg + NIVO 1 mg/kg Q3W, placebo to replace NIVO on weeks 3, 5, 9 and 11, monotherapy with NIVO 3 mg/kg Q2W to be continued during maintenance phase for 1 year after induction or disease progression	312	Recruiting	NA	NCT02523313
Treatment naïve unresectable/metastatic advanced melanoma	Phase 3	To determine the incidence of TRAE; Secondary outcomes: ORR and PFS (time frame: 20 weeks)	Arm 1: concomitant NIVO + IPI followed by monotherapy with NIVO; arm 2: sequential administration of NIVO and IPI, followed by monotherapy with NIVO	102	Recruiting	NA	NCT02905266
Stage III unresectable or stage IV melanoma	Phase 3	TRAE for up to 24 months; secondary outcomes: PFS (investigator assessed), OS, ORR	NIVO monotherapy or NIVO + IPI combination therapy	615	Recruiting	NA	NCT02599402; CheckMate 401
Stage III/IV melanoma	Phase 3 RCT	OS rate at the end of 2 years follow-up; secondary outcomes: PFS and RR	Arm A: induction with 2 courses of NIVO + IPI Q6W, maintenance with NIVO monotherapy Q6W for a total of 12 courses, patients cross over to arm C if disease progresses; arm D: induction with 2 courses of NIVO + IPI Q6W, maintenance with NIVO monotherapy Q6W for a total of 12 courses	300	Recruiting	NA	NCT02224781
Treatment naïve unresectable/metastatic advanced melanoma	Phase 3 RCT	To determine the incidence of TRAE; Secondary outcomes: ORR, PFS, OS	Arm 1: IPI 1 mg/kg + NIVO 3 mg/kg; arm 2: IPI 1 mg/kg + NIVO 6 mg/kg; arm 3: IPI 3 mg/kg + NIVO 1 mg/kg	340	Recruiting	NA	NCT02714218
Treatment naïve unresectable/metastatic advanced melanoma	Phase 3 RCT	OS (time frame: 44.1 months), PFS (time frame: up to 5 years)	Arm A: NIVO 3 mg/kg Q2W + placebo substituting IPI on weeks 1 and 4, followed by placebo substituting NIVO on week 4 for cycle 1 and 2; Arm B: 4 doses of NIVO 1 mg/kg + IPI 3 mg/kg Q3W followed by NIVO 3 mg/kg Q2W (placebo substituting NIVO on 3rd and 5th weeks for cycle 1 and 2; Arm C: 4 doses of IPI 3 mg/kg Q3W + placebo (replacing NIVO on 3rd and 5th weeks for cycles 1 and 2)	915	Completed, results available	OR: NIVO+IPI 58%, NIVO mono 44%, IPI mono 19%; CR: NIVO+IPI 19%, NIVO mono 16%, IPI mono 5%; Median PFS: NIVO monotherapy: 6.9 months (95%CI: 4.3 to 9.5), IPI monotherapy: 2.9 months (95%CI: 2.8 to 3.4) and IPI + NIVO: 11.5 months (95%CI: 8.9 to 16.7); median PFS for participants with PD-L1 positive malignancy: 14.0 months in NIVO arm and combination therapy arm; median PFS for participants with PD-L1 negative malignancy: 5.3 months (95%CI: 2.8 to 7.1) for NIVO monotherapy and 11.2 months (95%CI: 8.0 - not reached) for IPI + NIVO; OS at 2 years: NIVO+ IPI 64%, NIVO monotherapy 59% and IPI monotherapy 45%; OS at 3 years: NIVO+ IPI 58%, NIVO monotherapy 52% and IPI monotherapy 34%; median OS NIVO+ IPI-not reached (95%CI 38.2 months - not reached), NIVO monotherapy-37.6 months (95%CI: 29.1 - not reached) and IPI monotherapy-19.9 months (95%CI: 16.9–24.9); Grade 3–4 TRAEs: NIVO+IPI 59%, NIVO mono 21%, IPI mono 28%	NCT01844505; CheckMate 067 [56]

*Abbreviations: MTD* maximum tolerable dose, *DLT* dose limiting toxicity, *RCT* randomized controlled trial, *NA* not available, *CI* confidence interval, *ORR* overall response rate, *OS* overall survival, *PFS* progression free survival, *RR* response rate, *OR* objective response, *CR* complete response, *PR* partial response, *SD* stable disease, *PD* progressive disease, *TRAE* Treatment-related adverse events, *irAE* immune related adverse events, *OD* once daily dosing, *BID* twice daily, *QID* four times a day, *NIVO*, nivolumab, *IPI* ipilimumab, *PEMBRO* pembrolizumab, *RECIST* response evaluation criteria in solid tumors, *NCI CTCAE* National Cancer Institute common terminology criteria for adverse events, Q(x)W, every (x) weeks; D(x), day(x); *TRAE* treatment related adverse events, *AE* adverse events

**Table 2:** NSCLC trials evaluating combination checkpoint inhibition with anti-CTLA-4 plus anti- PD-1/PD-L1 monoclonal antibodies

Cancer type	Phase	Primary outcome	Dosing regimen	Enrollment number	Status	Results	Clinical trials identification number
Stage IV NSCLC	Phase 1	Evaluation of treatment related toxicity of specified treatment regimen in ALK/EGFR mutated NSCLC patients for up to 36 months; secondary outcomes: OS, PFS, RR evaluated up to 36 months	Arm 1 (EGFR mutant NSCLC): 4 doses of IPI 3 mg/kg + erlotinib 150 mg OD (or the tolerable dose); arm 2 (ALK positive NSCLC): 4 doses of IPI 3 mg/kg + crizotinib 250 mg BID (or tolerable dose); arm 3 (EGFR mutant NSCLC): erlotinib 150 mg OD (or tolerable dose) + NIVO 240 mg Q2W; arm D (ALK positive NSCLC): crizotinib 250 mg BID (or tolerable dose) + NIVO 240 mg Q2W	14	Active, not recruiting	NA	NCT01998126
Stage IIIb/IV NSCLC	Phase 1 RCT	Evaluate safety of specified treatment regimens; secondary outcomes: Evaluation of ORR and PFS rate (time frame: up to 24 weeks)	Arm A: gemcitabine + cisplatin and NIVO; arm B: pemetrexed + cisplatin and NIVO; arm C: carboplatin + paclitaxel and NIVO; arm D: NIVO + maintenance with bevacizumab; arm E: erlotinib + NIVO; arm F: NIVO; arm G (squamous NSCLC): IPI + NIVO; arm H (non-squamous NSCLC): IPI + NIVO; arm I (squamous NSCLC): IPI + NIVO; arm J (non-squamous NSCLC): IPI + NIVO; arm K (squamous NSCLC): NIVO; arm L (non-squamous NSCLC): NIVO; arm M (NSCLC with asymptomatic and untreated brain metastases): NIVO; arm N (NSCLC with any histology): IPI + NIVO; arm O: IPI + NIVO; arm P: IPI + NIVO; arm Q: IPI + NIVO; arm R: IPI + NIVO; arm S: IPI + NIVO	412	Completed	NIVO 3 mg/kg Q2W + IPI 1 mg/kg Q12W (*n* = 38): grade 3–4 TRAE: 37%, ORR: 47% (95%CI: 31–64), median PFS: 8.1 months (95%CI: 5.6–13.6), 1 year OS rate: not calculated; NIVO 3 mg/kg Q2W + IPI 1 mg/kg Q6W (*n* = 39): grade 3–4 TRAE: 33%, ORR: 39% (95%CI: 23–55), median PFS: 3.9 months (95%CI: 2.6–13.2), 1 year OS rate: 69% (95% CI: 52–81); NIVO 3 mg/kg Q2W (*n* = 52): grade 3–4 TRAE: 19%, ORR: 23% (95%CI: 13–37), median PFS: 3.6 months (95%CI: 2.3–6.6), 1 year OS rate: 73% (95% CI: 59–83); ORR for tumors having PD-L1 expression of ≥1% was reported as 57% and ORR for tumors having PD-L1 expression of ≥50% was reported as 92% with NIVO + IPI; ORR for tumors having PD-L1 expression of ≥1% was reported as 28% and ORR for tumors having PD-L1 expression of ≥50% was reported as 50% with NIVO monotherapy	NCT01454102; CheckMate 012 [60]
Advanced NSCLC	Phase 1b	OR assessed at 24 weeks, number of study participants experiencing DLT and number of participants that report treatment associated toxicities	Participants enrolled in dose escalation arm and all experimental arms receive MEDI4736 + tremelimumab combination therapy	747	Active, not recruiting, preliminary results available	OR in tremelimumab 1 mg/kg combination therapy cohort: Overall: 23% (6 of 26 patients, 95%CI: 9 to 44); PD-L1 positive tumors: 22% (2 of 9 patients, 95%CI: 3 to 60); PD-L1 negative tumors: 29% (4 of 14 patients, 95%CI: 8 to 58); MTD exceeded for therapy with tremelimumab 3 mg/kg + MEDI4736 20 mg/kg with DLT of 30% (2 of 6 patients)	NCT02000947 [62]
NSCLC with brain metastases	Phase 1/Phase 2	Recommended phase 2 dose of NIVO + stereotactic radiosurgery/ NIVO + whole brain radiation therapy/ NIVO + IPI and stereotactic radiosurgery/ NIVO + IPI and whole brain radiation therapy; the observed MTD from phase 1 to become the starting dose for phase 2	Experimental arm 1: NIVO 3 mg/kg Q2W + stereotactic surgery; experimental arm 2: NIVO 3 mg/kg Q2W + whole brain radiation therapy 30 Gy in 10 fractions; experimental arm 3: NIVO MTD as decided in phase 1 + IPI 1 mg/kg Q6W and stereotactic radiosurgery; experimental arm 4: NIVO MTD as decided in phase 1 + IPI 1 mg/kg (Q6W) and whole brain radiation therapy (30 Gy in 10 fractions)	80	Not yet recruiting	NA	NCT02696993
Unresectable/metastatic NSCLC	Phase 1/ Phase 2 RCT	Part I: determine recommended phase 2 dose of PEMBRO; part II: OR rate for cohort G and H; secondary outcomes: PFS, OS and duration of response (assessed up to 2 years)	Cohort H: IPI + PEMBRO (recommended phase 2 dose as per cohort D)	308	Recruiting, preliminary data available	Not available for cohort H.	NCT02039674; KEYNOTE 021 [61]
Advanced NSCLC	Phase 2	Best ORR evaluated Q6W up to 48 weeks	IPI 1 mg/kg Q6W + NIVO 3 mg/kg Q2W	35	Recruiting	NA	NCT02350764
Stage IV NSCLC	Phase 2	OR rate; secondary outcomes: PFS, duration of response (time frame: 6 months)	IPI + NIVO	590	Recruiting	NA	NCT02659059; CheckMate 568
Advanced NSCLC	Phase 2 RCT	PFS rate, ORR and duration of response (assessed up to 24 weeks)	Comparator arm: monotherapy with NIVO; arm 2: BMS-986016 + NIVO; arm 3: dasatinib + NIVO; arm 4: IPI + NIVO	504	Recruiting	NA	NCT02750514
Locally advanced/metastatic NSCLC	Phase 2 RCT	CR rate (assessed up to 2 years); secondary outcomes: ORR and disease control rate (assessed up to 2 years)	Arm 1: gefitinib + MEDI4736; arm 2: AZD9291 + MEDI4736; arm 3: docetaxel + selumetinib and MEDI4736; arm 4: tremelimumab + MEDI4736	49	Completed	Not yet available	NCT02179671
Recurrent Stage IV squamous cell lung carcinoma	Phase 2/ Phase 3 RCT	ORR, OS (assessed for 3 years), IA-PFS (evaluated for 18 months), IA-PFS and OS in study participants receiving experimental regimen versus standard of care; secondary outcomes: duration of response, RR, PFS and OS for experimental regimen, frequency of toxicity events (assessed for 3 years)	S1400I arm I: IPI + NIVO; S1400I arm II: NIVO monotherapy	10,000	Recruiting	NA	NCT02154490; lung-MAP trial [79]
Stage IV/recurrent NSCLC	Phase 3	Percent study participants with high grade toxicity; secondary outcomes: PFS, ORR, duration of response (assessed for 40 months)	NIVO + IPI	1500	Recruiting	NA	NCT02869789
NSCLC	Phase 3 RCT	Major pathological response rate (determined at surgery); secondary outcomes: complete pathological response rate (determined at surgery), OS and event free survival assessed for up to 130 months	Experimental arm: IPI + NIVO	326	Not yet recruiting	NA	NCT02998528
Stage IV squamous cell carcinoma	Phase 3 RCT	OS (time frame: 3 years), IA-PFS (time frame: 18 months)	Active comparator: NIVO on D1, repeated every 14 days; experimental arm: NIVO + IPI D1 of every 3rd course, course to be repeated every 14 days	350	Recruiting	NA	NCT02785952
Chemotherapy naïve/recurrent stage IV NSCLC	Phase 3 RCT	OS, assessed up to 48 months; PFS, assessed up to 40 months; Secondary outcome: OR rate, assessed up to 48 months	Arm A: NIVO monotherapy; arm B: IPI + NIVO combination therapy; arm C: platinum doublet chemo (carboplatin/ cisplatin + gemcitabine for squamous histology and carboplatin/ cisplatin + pemetrexed for non-squamous histology) + NIVO	2220	Recruiting	NA	NCT02477826; CheckMate 227
Stage IV/recurrent NSCLC	Phase 3 RCT	PFS for T790 M negative, EGFR positive NSCLC, evaluated for 33 months; secondary outcomes: PFS rate, ORR and duration of response evaluated for 33 months; OS assessed for 5 years	Arm 1: IPI + NIVO; arm 2: platinum doublet therapy (cisplatin/ carboplatin + pemetrexed) + NIVO	465	Recruiting	NA	NCT02864251; CheckMate 722
Advanced/metastatic NSCLC	Phase 3 RCT	OS with MEDI4736 + tremelimumab combination versus standard of care treatment (evaluated for 4 years)	Arm 1: MEDI4736 + tremelimumab	800	Recruiting	NA	NCT02542293; NEPTUNE study
Locally advanced/metastatic NSCLC	Phase 3 RCT	PFS and OS assessed up to 3 years; secondary outcomes: ORR (evaluated for 3 years), proportion of study participants alive at end of 1 year of randomization and duration of response (evaluated for 3 years)	Sub-study-A experimental arm: participants with PD-L1 positive malignancy to receive MEDI4736; sub-study B experimental arm 1: participants with PD-L1 negative malignancy to receive MEDI4736 + tremelimumab; sub-study experimental arm 2: participants with PD-L1 negative malignancy to receive MEDI4736 only; sub-study B experimental arm 3: participants with PD-L1 negative malignancy to receive tremelimumab only	730	Active, not recruiting	NA	NCT02352948; ARCTIC study
Advanced/metastatic NSCLC	Phase 3 RCT	OS and PFS with MEDI4736 + tremelimumab combination versus standard of care treatment (evaluated for up to 3 years)	Arm 1: MEDI4736 monotherapy; arm 2: MEDI4736 + tremelimumab	1092	Active, not recruiting	NA	NCT02453282; MYSTIC study

*Abbreviations: NSCLC* non-small cell lung cancer, *EGFR* epithelial growth factor receptor, *ALK* anaplastic lymphoma kinase, *MTD* maximum tolerable dose, *DLT* dose limiting toxicity, *RCT* randomized controlled trial, *NA* not available, *OR* objective response, *CR* complete response, *CI* confidence interval, *TRAE* treatment-related adverse events, *NIVO* nivolumab, *IPI* ipilimumab, *PEMBRO* pembrolizumab, MEDI4736, durvalumab, *ORR* overall response rate, *OS* overall survival, *PFS* progression free survival, *RR* response rate, *OD* once daily dosing, *BID* twice daily, Q(x)W, every (x) weeks; D(x), day(x)

### Combination checkpoint inhibition trials in melanoma

Massive efforts are testing dual checkpoint inhibition with anti-PD-1/PD-L1 plus anti-CTLA-4 MoAbs in patients with melanoma. Presently, there are 28 clinical trials in various phases committed to this objective (Table 1). Currently, only one trial is in the process of recruiting participants that will receive ipilimumab in combination with pembrolizumab. Approximately two-thirds of all ongoing trials (18 of 28 trials) in melanoma patients are investigating combined therapy with nivolumab plus ipilimumab.

Several distinct methodologies for the combined use of nivolumab and ipilimumab have been identified and are presently being investigated for potential clinical benefit in two trials. One trial will compare the incidence of adverse events, overall response rate (ORR), and progression free survival (PFS) with concurrent versus sequential administration of nivolumab and ipilimumab in the induction phase (NCT02905266). Another trial, CheckMate 064, will evaluate the duration of response, response rate (RR), rate of progression and the proportion of study participants that develop grade 3–5 adverse events when using nivolumab prior to ipilimumab versus ipilimumab prior to nivolumab in the induction phase (NCT01783938).

Treatment protocols in five trials involve the integrated use of nivolumab and/or ipilimumab with additional anti-cancer agents. The assessment of MTD and ORR for combined therapy with nivolumab plus ipilimumab and ACY241 (histone deacetylase {HDAC} inhibitor) will be performed in one trial (NCT02935790) [10]. Another trial will be exploring the clinical efficacy and incidence of adverse events for nivolumab plus TAK580 (pan-Raf kinase inhibitor), nivolumab and plozalizumab (chemokine receptor-2 {anti-CCR-2} MoAb), and the combined use of nivolumab plus ipilimumab and vedolizumab (anti-LPAM-1/α4β7 MoAb), each in separate arms (NCT02723006) [10]. Similarly, a different trial will evaluate the safety profile and clinical efficacy with combination dabrafenib (BRAF protein inhibitor) and/or trametinib (MEK MAPK/ERK kinase {mitogen-activated protein kinase kinase} inhibitor) in the induction phase, followed by ipilimumab and/or nivolumab (NCT01940809) [10]. This trial will also compare the outcomes of the above treatment regimens with nivolumab plus ipilimumab (NCT01940809). The SECOMBIT study will compare survival outcomes among several variations of treatment regimens composed of binimetinib (MEK1/2 inhibitor), encorafenib (Raf kinase inhibitor), nivolumab and ipilimumab (NCT02631447) [10]. Lastly, one trial will analyze response to vemurafenib (BRAF-V600 kinase inhibitor) plus cobimetinib (MAP2K1/MEK1 inhibitor) in the induction phase followed by nivolumab plus ipilimumab, versus nivolumab plus ipilimumab combination therapy alone (NCT02968303) [10].

Several additional treatment modalities with nivolumab plus ipilimumab combination therapy for melanoma is being examined in three trials. Neoadjuvant therapy with nivolumab and combined nivolumab plus ipilimumab is under evaluation in NCT02519322 and NCT02977052, respectively. A different trial will assess the use of Yttrium-90 selective internal hepatic radiation prior to induction with nivolumab plus ipilimumab (NCT02913417).

The safety and survival statistics from five clinical trials that applied combined immunotherapy with various anti-PD-1/PD-L1 plus anti-CTLA-4 MoAbs has been made available. One trial enrolled 136 patients with unresectable stage III/IV malignant melanoma in eight cohorts (NCT01024231). Each cohort received a unique regimen, differing in sequence of administration and dosing for nivolumab and ipilimumab. The doses for nivolumab were 1, 3, or 10 mg/kg whereas ipilimumab was administered at 3 or 10 mg/kg, per predefined criteria for each cohort. The study documented a considerable variation in OS among different cohorts. In one group, among 14 patients that received nivolumab 0.3 mg/kg plus ipilimumab 3 mg/kg, the 1 year OS rate was 56%, whereas among six patients receiving nivolumab 3 mg/kg plus ipilimumab 3 mg/kg the 1 year OS rate was 100% [54]. Another parameter compared in this study was median OS for concurrent and sequential administration of nivolumab and ipilimumab, which was noted to be 39.7 months and 13.0 months, respectively. The study also recorded objective response stratified on the basis of PD-L1 status. In patients with PD-L1 positive melanoma, an objective response was noted in 6 of 13 participants receiving concurrent therapy and 4 of 8 participants receiving sequential therapy. In the PD-L1 negative subgroup 9 of 22 patients responded with concurrent therapy and 1 of 13 patients receiving sequential therapy responded. Treatment related adverse events were noted in 93% (grade 3–4 adverse events: 53%) patients receiving the concurrent regimen and 73% (grade 3–4 adverse events: 18%) in the sequential therapy group [54].

The outcomes for combined therapy with nivolumab plus ipilimumab versus placebo plus ipilimumab in *BRAF*-wild type and *BRAF*-mutant unresectable/metastatic melanoma were recorded in one trial (CheckMate 069, NCT01927419). Combination therapy appeared to be effective in patients with both *BRAF*-wild type (objective response: 61%, 44 of 72 patients, 95%CI: 49 to 72; complete response: 22%, 16 patients) and *BRAF*-mutant tumors (objective response: 52%, 12 of 23 patients, 95%CI: 31 to 73; complete response: 22%, 5 patients) [53]. The placebo group, however, exhibited poor outcomes in both *BRAF*-wild type (objective response: 11%, 4 of 37 patients, 95%CI: 3 to 25; complete response: 0%) and *BRAF* mutant tumors (objective response: 10%, 1 of 10 patients, 95%CI: 0 to 45; complete response: 0%). A subgroup analysis in this study evaluated the influence of tumor PD-L1 status on clinical outcomes. Patients receiving combination therapy demonstrated no significant difference in objective response rates (PD-L1 positive: 58, 95%CI: 37–78; PD-L1 negative: 55, 95%CI: 41–69) [53].

Another trial assessing survival outcomes for combined therapy with nivolumab plus ipilimumab versus placebo in treatment naïve unresectable/metastatic melanoma reported a median PFS for nivolumab monotherapy as 6.9 months (95%CI: 4.3 to 9.5), ipilimumab monotherapy as 2.9 months (95%CI: 2.8 to 3.4), and combined therapy with ipilimumab plus nivolumab as 11.5 months (95%CI: 8.9 to 16.7) (CheckMate 067, NCT01844505) [55, 56]. The study reported an overall survival at 2 years for nivolumab plus ipilimumab combination as 64%, nivolumab monotherapy as 59% and ipilimumab monotherapy as 45%. At 3 years follow-up, these were 58, 52 and 34%, respectively. Median overall survival for ipilimumab monotherapy was recorded as 19.9 months (95%CI: 16.9–24.9), nivolumab monotherapy as 37.6 months (95%CI: 29.1 - not reached) and as not reached (95%CI 38.2 months - not reached) for combination therapy arm. The objective response with combination therapy was noted in 58% patients, 19% with ipilimumab monotherapy and 44% with nivolumab monotherapy whereas complete response was observed in 19, 5 and 16% patients, respectively [56]. This data from CheckMate 067 allowed for confirmatory approval of combined therapy with nivolumab and ipilimumab for melanoma that initially received an accelerated approval by the FDA based upon the findings of CheckMate 069 trial. In addition to the above, the CheckMate 067 study also presented data on the influence of PD-L1 status on clinical outcomes. Patients with PD-L1 positive melanoma that received either combination therapy or nivolumab monotherapy had a median PFS of 14.0 months. However, study participants with PD-L1 negative melanoma exhibited a median PFS of 5.3 months (95%CI: 2.8 to 7.1) with nivolumab monotherapy and 11.2 months (95%CI: 8.0 - not reached) with combination therapy [55]. Lastly, with regards to the safety, this trial noted treatment related grade 3–4 adverse events in 59% patients receiving nivolumab plus ipilimumab therapy, 21% of those receiving nivolumab only and 28% patients treated with ipilimumab monotherapy [56].

A different trial investigating the effects of variation in the sequence of administering different immunotherapy agents observed that the patient cohort receiving nivolumab before ipilimumab exhibited grade 3–5 treatment related adverse events (TRAE) in 50% patients, response at 25 weeks in 41% and 12 months overall survival in 76% patients (CheckMate 064, NCT01783938) [57]. In the patient cohort receiving ipilimumab prior to nivolumab, 42.8% patients had a grade 3–4 TRAE, 20% patients exhibited response at 25 weeks and 12 months overall survival was noted in 54% patients [57].

Finally, one trial investigated the safety and efficacy of combined therapy with reduced dose ipilimumab (1 mg/kg every 3 weeks for 4 doses) plus full-dose pembrolizumab (2 mg/kg every 3 weeks) in patients with advanced melanoma (KEYNOTE-029, NCT02089685) [58]. This trial documented grade 1–2 and grade 3–4 treatment related adverse events in 51% (78 of 153) and 45% (69 of 153) patients, respectively. Immune-related adverse events were reported in 60% (92 of 153) of melanoma patients, of which 27% (42 of 153) patients experienced one or more grade 3–4 immune-related adverse events. A number of study participants discontinued treatment due to treatment related adverse events. This included 14% (22 of 153) patients discontinuing both ipilimumab and pembrolizumab, 9% (14 of 153) patients stopping pembrolizumab only and 8% (12 of 153) patients that discontinued ipilimumab only. With respect to survival outcomes (median 17.0 months follow-up), the study reported a PFS at 12 months of 69% (95%CI: 60–75), objective response of 61% (93 of 153 patients, 95%CI: 53–69), complete response of 15% (23 of 153 patients), partial response of 46% (70 of 153 patients), stable disease in 18% (28 of 153 patients) and progressive disease in 19% (29 of 153 patients) [58]. These results were considerably better in terms of survival benefit and toxicity profile than combination therapy with nivolumab and ipilimumab for melanoma. Combined checkpoint inhibition with nivolumab 1 mg/kg plus ipilimumab 3 mg/kg evaluated in early phase 1 trials reported an objective response rate of 53% (9 of 17 patients, 95%CI: 28 to 77), complete response of 17% (3 of 17 patients), partial response of 35% (6 of 17 patients) and serious treatment related adverse events in 49% patients (NCT01024231) [54, 59].

### Combination checkpoint inhibition trials in NSCLC

Combined immunotherapy with anti-PD-1/PD-L1 and anti-CTLA-4 MoAbs in patients with NSCLC is currently being investigated in 16 ongoing trials (Table 2). Over half (10 of 16 trials) of these trials are evaluating combination therapy with nivolumab and ipilimumab with or without other therapeutic modalities. Other combination checkpoint inhibition regimens that are being examined in patients with NSCLC include tremelimumab plus durvalumab (5 of 16 trials) and pembrolizumab plus ipilimumab (1 of 16 trials).

Presently, five ongoing trials are assessing the combined use of anti-PD-1/PD-L1 and/or anti-CTLA-4 MoAbs with various chemotherapy agents. One of the trials has defined treatment cohorts on the basis of patient tumor marker status (NCT01998126). Patients with *EGFR* mutant NSCLC will receive erlotinib with either nivolumab or ipilimumab and those with *ALK* rearranged NSCLC will be administered crizotinib with either nivolumab or ipilimumab. Another trial will compare PFS, ORR, and duration of response in patients with advanced NSCLC after administration of dasatinib (SRC-family protein-tyrosine kinase inhibitor) plus nivolumab, BMS-986016 (anti- LAG-3 {lymphocyte activation gene-3} MoAb) plus nivolumab and ipilimumab plus nivolumab (NCT02750514) [10]. Participants enrolled in CheckMate 227 will be randomized to receive nivolumab plus platinum doublet chemotherapy (carboplatin/cisplatin plus gemcitabine for squamous NSCLC and carboplatin/cisplatin plus pemetrexed for non-squamous NSCLC) or combined therapy with nivolumab plus ipilimumab (NCT02477826). Similarly, CheckMate 722 will evaluate PFS for T790 M negative, *EGFR* mutant NSCLC patients treated with nivolumab plus platinum doublet chemotherapy (carboplatin/cisplatin plus pemetrexed) and ipilimumab plus nivolumab combination therapy (NCT02864251). The KEYNOTE 021 trial will focus on determining the RP2D for pembrolizumab plus ipilimumab, and it has a planned survival assessment to follow (NCT02039674). This trial will also evaluate the combined use of pembrolizumab with one or more standard chemotherapy agents using pre-defined treatment protocols. These include carboplatin, pemetrexed, paclitaxel, bevacizumab, erlotinib, and gefitinib (NCT02039674).

The development of a treatment regimen that integrates immunotherapy with PD-1/PD-L1 and CTLA-4 checkpoint inhibitors with surgery and radiation has been undertaken in one trial (NCT02696993). This trial will determine the RP2D for four combination therapies in NSCLC patients with brain metastases at the time of enrollment. The treatment regimens specified by the study protocol include nivolumab plus stereotactic radiosurgery, nivolumab plus ipilimumab and whole brain radiation therapy, nivolumab plus whole brain radiation therapy, and nivolumab plus ipilimumab and stereotactic radiosurgery.

At present, data on safety and survival benefit from combined immunotherapy with PD-1/PD-L1 and CTLA-4 checkpoint inhibitors in NSCLC is available from three trials. One trial evaluated four experimental dosing schedules of combined therapy with nivolumab and ipilimumab against one monotherapy arm (nivolumab) in order to identify the regimen that delivers maximum clinical benefit with an acceptable adverse-effects profile (CheckMate 012, NCT01454102). In the patient cohort receiving ipilimumab 1 mg/kg every 6 weeks plus nivolumab 3 mg/kg every 2 weeks an ORR of 39% was observed (95%CI: 23–55), median PFS was 3.9 months (95%CI: 2.6–13.2), the 1 year OS rate was 69% (95% CI: 52–81), and grade 3–4 treatment related adverse events (TRAE) occurred in 33% of patients [60]. In another cohort, designated to receive ipilimumab 1 mg/kg every 12 weeks plus nivolumab 3 mg/kg every 2 weeks, reported grade 3–4 TRAE occurred in 37% of patients, the ORR was 47% (95%CI: 31–64), median PFS was 8.1 months (95%CI: 5.6–13.6), and the 1 year OS rate was not calculated. Alternatively, patients treated with nivolumab 3 mg/kg every 2 weeks were noted to have an ORR of 23% (95%CI: 13–37), median PFS of 3.6 months (95%CI: 2.3–6.6), a 1 year OS rate of 73% (95% CI: 59–83), and TRAE occurred in 19% of patients. The study also attempted to correlate the data for treatment response to tumor PD-L1 expression. In patients treated with nivolumab plus ipilimumab combination therapy, the ORR for tumors with PD-L1 expression ≥1% and ≥ 50% were 57 and 92%, respectively. Conversely, patients receiving nivolumab monotherapy experienced an ORR of 28 and 50% for tumors with PD-L1 expression of ≥1% and ≥ 50%, respectively. Based on the overall analysis, treatment with ipilimumab 1 mg/kg every 6 weeks plus nivolumab 3 mg/kg every 2 weeks was selected for further investigation [60]. This regimen is presently under evaluation in the CheckMate 227 trial (NCT02477826).

The KEYNOTE 021 trial evaluated the combined use of pembrolizumab and traditional chemotherapy; it noted an ORR of 52% (13 of 25 patients, 95%CI: 31 to 72) and PFS of 10 months (95%CI: 4 - not reached) in patients treated with carboplatin 6 mg/mL/min plus pembrolizumab 2 or 10 mg/kg and paclitaxel 200 mg/m^2^ (NCT02039674) [61]. Likewise, the ORR was 48% (12 of 25 patients, 95%CI: 28 to 69) and PFS was “not reached” (95%CI: 4 - not reached) in patients that received paclitaxel 200 mg/m^2^ plus pembrolizumab 2 or 10 mg/kg, carboplatin 6 mg/mL/min and bevacizumab 15 mg/kg. Another cohort of patients that were given pemetrexed 500 mg/m^2^ plus carboplatin 5 mg/mL/min and pembrolizumab 2 or 10 mg/kg were noted to have an ORR of 71% (17 of 24 patients, 95%CI: 49 to 87) and PFS of 10 months (95%CI: 6 to 15) [61].

A different trial assessing combination checkpoint inhibition in patients with NSCLC involved the use of various doses of tremelimumab (1, 3 or 10 mg/kg) in combination with durvalumab (3, 10, 15 or 20 mg/kg) during the dose escalation phase (NCT02000947). The objective response (investigator assessed) in the tremelimumab 1 mg/kg treatment cohort was 23% (6 of 26 patients, 95%CI: 9 to 44) [62]. Also, an objective response was noted in 22% (2 of 9 patients, 95%CI: 3 to 60) of cases with PD-L1 positive tumors and 29% (4 of 14 patients, 95%CI: 8 to 58) of cases with PD-L1 negative tumors. MTD was exceeded for treatment with tremelimumab 3 mg/kg plus durvalumab 20 mg/kg, with almost 30% (2 of 6 patients) of patients reporting DLTs [62].

## Discussion

There is great enthusiasm surrounding combination immunotherapy with PD-1/PD-L1 and CTLA-4 checkpoint inhibitors. The superior outcomes with combined immunotherapy over single-agent regimens in preclinical studies, together with the approval of nivolumab plus ipilimumab combination therapy for patients with melanoma have shed light on the therapeutic potential of this concept. The possibility of expanding the spectrum of indications for combination checkpoint inhibition to a wide range of tumor histologies is being explored in several trials. Simultaneously, extensive efforts have been undertaken to optimize clinical benefit to adverse effects ratios with combination checkpoint inhibition.

**Fig. 3:**
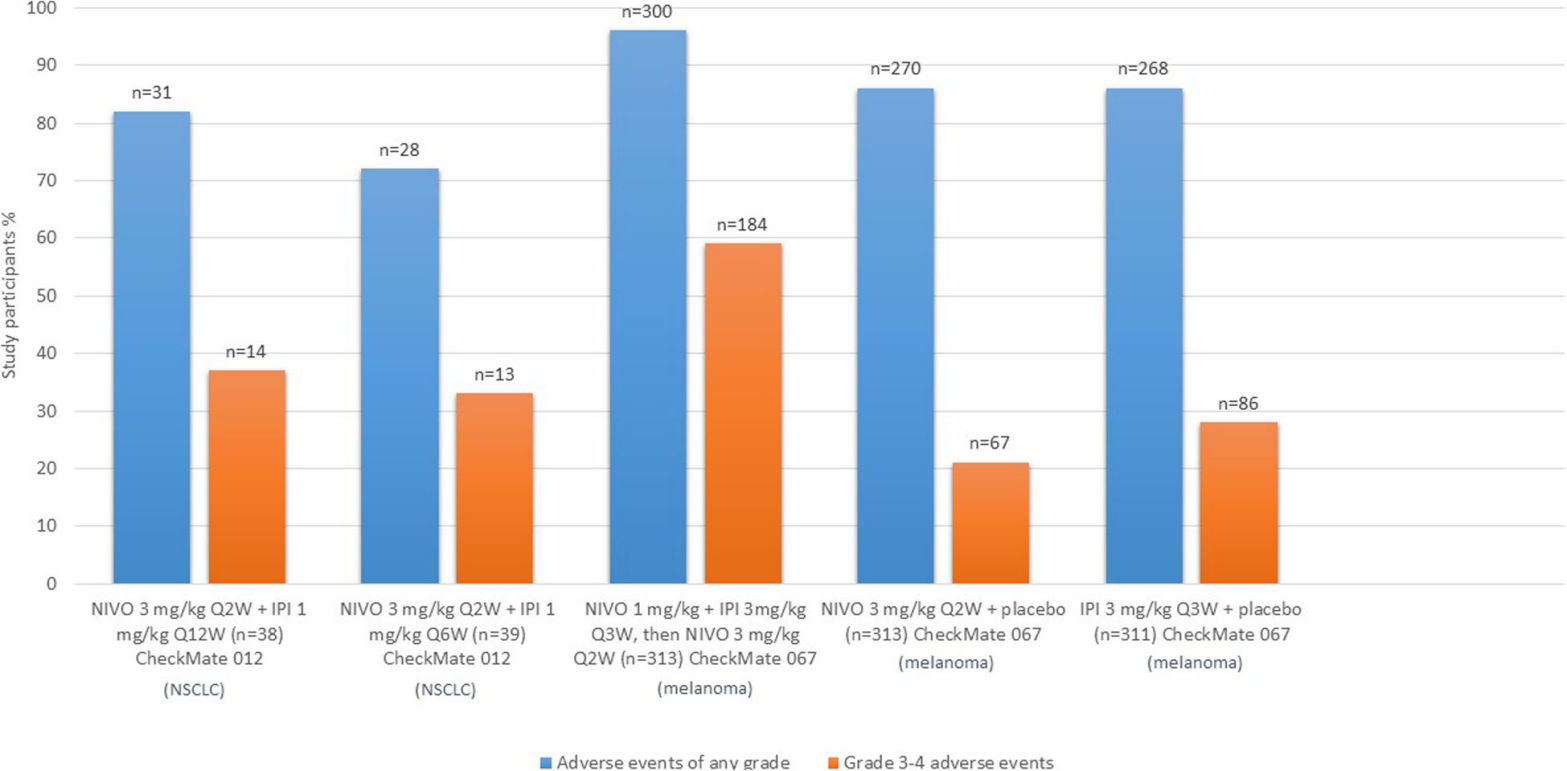
Comparison of objective response in select trials. Legend: Figure presenting a comparison of objective response in study participants, expressed in percent individuals, with different treatment regimens in CheckMate 012, NCT02000947 and CheckMate 067 trials. Abbreviations: NIVO, nivolumab; IPI, ipilimumab; DURVA, durvalumab; TREME, tremelimumab; Q(x)W, every (x) weeks; NSCLC, non-small cell lung cancer

Combined therapy with anti-PD-1/PD-L1 and anti-CTLA-4 MoAbs in advanced melanoma has exhibited better survival outcomes in comparison with single-agent immunotherapy (Fig. 3). CheckMate 067 reported a survival benefit with nivolumab plus ipilimumab combination therapy (median PFS: 11.5 months, objective response 58%) over monotherapy with ipilimumab (median PFS: 2.9 months, objective response 19%) and nivolumab (median PFS: 6.9 months, objective response 44%) [55, 56, 63]. It is, however, important to note that though this study met the co-primary end-point of exhibiting improved overall survival with combination therapy versus ipilimumab, it was underpowered to reflect upon the use of nivolumab and ipilimumab combination therapy over anti-PD-1 monotherapy followed by subsequent ipilimumab rescue. Another trial, CheckMate 069, recorded the hazard ratio for death or progression of disease for combined versus single-agent immunotherapy for *BRAF* wild-type as 0.40 (95% CI: 0.23 to 0.68, *p* < 0.001) and *BRAF*-mutant as 0.38 (95% CI: 0.15 to 1.00) melanoma patients, underscoring the survival benefit with combination checkpoint inhibition [53]. Akin to the aforementioned trials, several ongoing trials will compare single-agent immunotherapy with combination checkpoint inhibition with anti-PD-1/PD-L1 plus anti-CTLA-4 checkpoint inhibitors (NCT01940809, NCT02736123, NCT02519322, NCT02731729, NCT02374242, NCT02523313, NCT02599402, NCT02460068, NCT02750514, NCT02154490, NCT02785952, NCT02477826, NCT02352948, NCT02453282, NCT01928394, NCT02537418).

Several studies have attempted to correlate the expression of PD-L1 and response to combination checkpoint inhibition with anti-PD-1/PD-L1 plus anti-CTLA-4 MoAbs. PD-L1 positivity has classically been defined as visualization of at least 5% of tumor cells with PD-L1 staining in a section containing a minimum of one hundred cells suitable for evaluation [53, 54, 64, 65]. NCT01024231 documented response with combination therapy regardless of tumor PD-L1 status [54]. Likewise, CheckMate 069 reported no significant variation in response to combination checkpoint inhibition based on PD-L1 status [53]. Interestingly, patients with PD-L1 positive melanoma had better objective response rates as compared to those who tested negative for PD-L1 with ipilimumab monotherapy [53]. Another trial, CheckMate 067, noted a considerable advantage in median PFS with combined ipilimumab plus nivolumab (11.2 months) as opposed to nivolumab monotherapy (5.3 months) in patients with PD-L1 negative melanoma. The median PFS for patients with PD-L1 positive tumors, however, was the same for both treatment groups (14 months) [65]. Similarly, a different trial evaluating low-dose ipilimumab plus nivolumab in NSCLC patients reported an ORR of 57% in patients with tumor PD-L1 expression ≥1 and 92% in patients with tumor PD-L1 expression ≥50% (CheckMate 012) [60]. Considering the above, it appears that a pragmatic approach for selecting the most appropriate immunotherapy regimen (monotherapy versus combination therapy) may be through a novel treatment algorithm including tumor PD-L1 status.

The incidence of treatment-related adverse events with combination checkpoint inhibition has been a matter of concern in pivotal trials. The CheckMate 069 trial, which evaluated combination therapy with nivolumab and ipilimumab (nivolumab 1 mg/kg plus ipilimumab 3 mg/kg every 3 weeks for 4 doses followed by monotherapy with nivolumab 3 mg/kg every 2 weeks) versus ipilimumab monotherapy in melanoma patients, documented treatment-associated grade 3–4 adverse events in 54% of patients receiving combination therapy as compared to 24% receiving ipilimumab monotherapy [53]. These results suggest that melanoma patients receiving combination checkpoint inhibition were much more likely to develop severe drug-related adverse events as compared to treatment with ipilimumab monotherapy. Although 68% of patients that discontinued combination therapy due to toxicity exhibited an objective response, concerns over treatment-related toxicity with combination checkpoint inhibition regimens persuade some to favor immune-checkpoint inhibitor monotherapy [53]. In order to validate these findings, the same combination therapy regimen was evaluated in the CheckMate 067 trial and compared ipilimumab monotherapy and nivolumab monotherapy in parallel arms [56]. The frequency of grade 3–4 adverse events in patients treated with combination therapy (59% patients) was higher than that recorded for patients receiving monotherapy with ipilimumab (28% patients) or nivolumab (21% patients) [56]. However, treatment related adverse events with combination therapy were described as manageable and the study concluded that this regimen was suitable for further investigation. Considering the above, it may be stated that one should be cautious in selection of combination immune checkpoint inhibition over monotherapy in elderly patients with high frailty index.

A retrospective pooled analysis conducted to study efficacy and safety of combined therapy with nivolumab and ipilimumab in patients that discontinued therapy due to adverse events presented interesting findings. Data from phase 2 and phase 3 trials for advanced melanoma evaluating nivolumab 1 mg/kg plus ipilimumab 3 mg/kg every 3 weeks followed by monotherapy with nivolumab 3 mg/kg every 2 weeks, was pooled to compare outcomes in participants who discontinued therapy due to adverse events versus those who did not [66]. After a minimum of 18 months follow-up, median PFS and objective response rate for patients that discontinued therapy during induction were found to be 8.4 months and 58.3% respectively. On the other hand, patients that did not discontinue therapy were found to have a median PFS and objective response rate of 10.8 months and 50.2%, respectively. The nearly similar efficacy outcomes in the two groups indicated that despite discontinuing the therapy, patients continued to derive benefits from the treatment [66]. In other words, shorter course of treatment exhibited outcomes comparable to those achieved with full-course combination therapy. Also, it may be argued that immune related adverse events could possibly serve as surrogate markers for patients that may benefit from immunotherapy regimens.

**Fig. 4:**
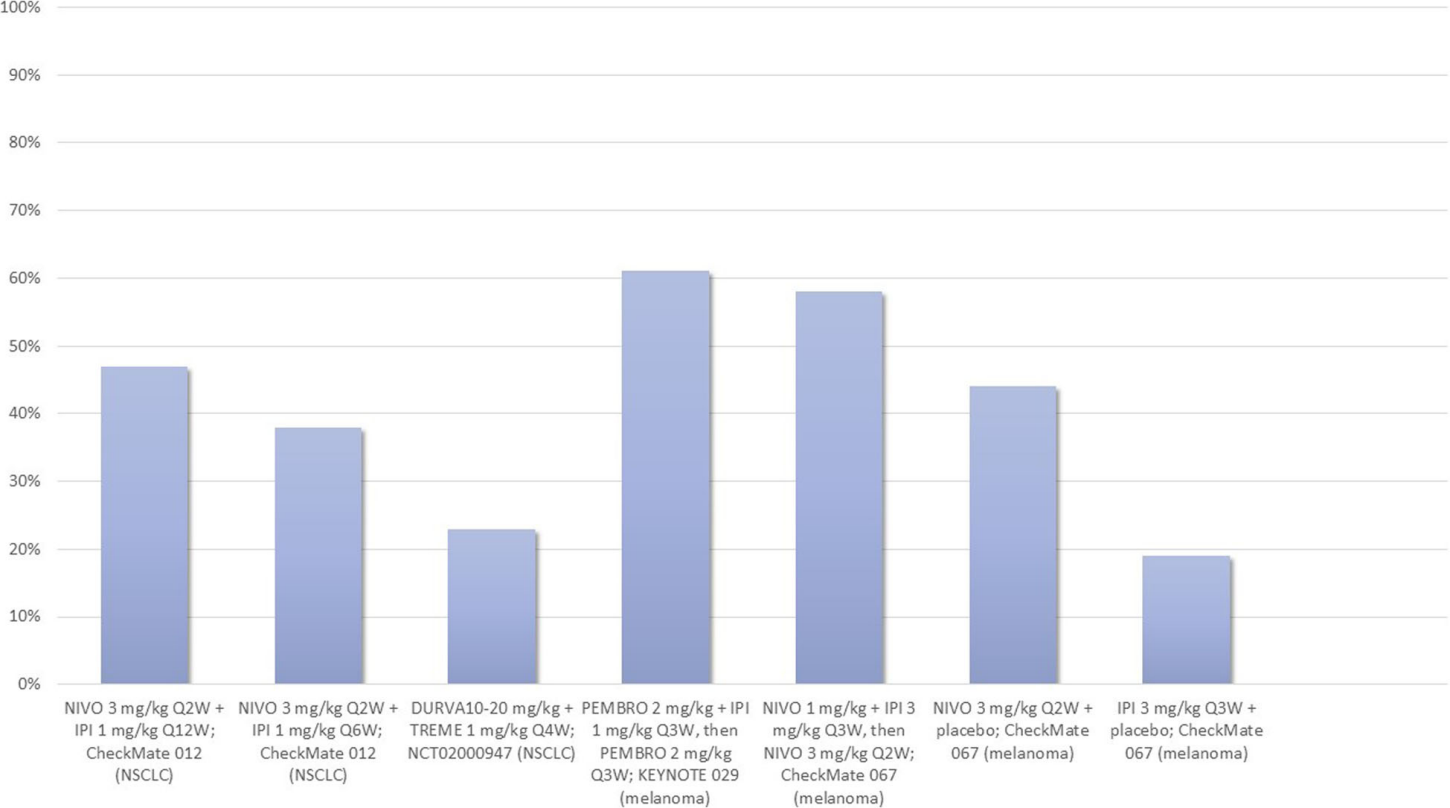
Comparison of treatment related adverse events for different regimens in CheckMate 012 and CheckMate 067. Legend: The figure elaborates percent patients documented to have adverse events with different treatment regimens assessed in the CheckMate 012 and CheckMate 067 trials. Please note that some participants had more than one adverse event. No treatment associated deaths were reported till point of assessment in CheckMate 012 while 2 treatment related deaths in CheckMate 067 nivolumab group were reported > 100 days after discontinuation of therapy. Abbreviations: NIVO, nivolumab; IPI, ipilimumab; n, number of patients; X axis: treatment regimens, Y axis: percent patients relative to cohort size

The use of low-dose combination checkpoint inhibition appears to be a promising approach for improving clinical benefit without significantly increasing adverse events. The CheckMate 012 trial assessed several nivolumab plus ipilimumab combination regimens as first-line therapy in NSCLC patients. The frequency of treatment-associated grade 3–4 adverse events with nivolumab 3 mg/kg every 2 weeks plus ipilimumab 1 mg/kg every 12 weeks was 37% and with nivolumab 3 mg/kg every 2 weeks plus ipilimumab 1 mg/kg every 6 weeks was 33% [60]. The frequency of treatment-related grade 3–4 adverse events with nivolumab monotherapy was 19% [60]. Low-dose combination checkpoint inhibition with nivolumab and ipilimumab had a more acceptable adverse-effects profile compared to the combination regimen evaluated in CheckMate 067 (Fig. 4). However, it may be argued that the variation in toxicity profile was secondary to, or at least in part influenced by a difference in tumor histologies. One example may be the FDA approved regimen for melanoma (4 cycles of ipilimumab 3 mg/kg plus nivolumab 1 mg/kg every 3 weeks followed by nivolumab 240 mg every 2 weeks), that exhibited an acceptable safety profile and response in recurrent small cell lung cancer but at the same time demonstrated a poor safety and efficacy profile in NSCLC, leading to abandonment of this strategy in the latter [60, 67]. Data from ongoing studies investigating low-dose combination checkpoint inhibition in a number of tumor histologies will be crucial to the validation of these findings.

Another step towards achieving the right balance of efficacy and incidence of adverse events may be to critically assess the use of common terminology criteria for adverse events (CTCAE). CTCAE are used for documenting chemotherapy associated adverse events [68]. They are instrumental to determine the appropriate dose limiting toxicity for the experimental regimen in a trial. This in-turn has a significant bearing on the recommended phase 2 dose for the novel therapeutic agent. CTCAE are, however, now being applied to immune related adverse events for novel immunotherapy regimens [69]. A phase 1 trial investigating nivolumab plus ipilimumab in melanoma patients used an asymptomatic rise in lipase as the primary dose limiting toxicity, central to informing the recommended phase 2 dose in this trial. A retrospective study analyzed the association between asymptomatic rise in lipase and amylase (grade 3 and above) with pancreatitis in 119 participants and found only 2 patients to have pancreatitis. This represented 6.3% of all patients with grade 3 and above lipase and 20% of those with grade 3 or above increase in both amylase and lipase. Thus, in simple terms, lipase did not appear to be a relevant marker for pancreatitis. This observation thus signifies the need to exercise appropriate caution when grading independent lab values using CTCAE in immunotherapy trials [69].

Existing evidence suggests that single-agent immunotherapy for tumors with high PD-L1 expression (≥50% cells positive for PD-L1 staining) can achieve far superior outcomes than chemotherapy in similar settings. In patients with NSCLC with high PD-L1 expression, pembrolizumab exhibited a response rate of 45%, PFS of 10.3 months, and a 1-year survival rate of 70% [70]. In comparison, treatment with standard of care chemotherapy had a response rate of 28%, PFS of 6 months, and 1-year survival rate of 54% (KEYNOTE 026) [70]. On the other hand, trials involving a lower cut-off value for PD-L1 positivity (≥5% cells positive for PD-L1 staining) failed to demonstrate any advantage in clinical efficacy with single-agent immunotherapy over standard chemotherapy (CheckMate 026) [71, 72]. Reflecting on the data on efficacy and overall toxicity profile of single-agent immunotherapy regimens in tumors with high PD-L1 expression, outperforming these regimens may prove to be a challenge for combination checkpoint inhibition regimens. Further data from ongoing trials will be vital to conclusively determine if single-agent immunotherapy with pembrolizumab can be replaced with a combined immunotherapy regimen.

Immunotherapy with PD-1/PD-L1 plus CTLA-4 checkpoint inhibitors for a diverse set of solid tumors is currently being investigated in 8 trials (Table 3). Half of all phase 1/phase 2 solid tumor trials are evaluating combined therapy with nivolumab and ipilimumab. Others include three trials with combined therapy with tremelimumab plus durvalumab and one trial with atezolizumab plus ipilimumab. Each of these eight trials will evaluate combination checkpoint inhibition regimens in a large number of malignancies. The data gathered from these studies will be crucial to identifying tumor histologies that would benefit most from combination checkpoint inhibition.

**Table 3:** Phase 1 solid tumor trials investigating combined immunotherapy with anti-CTLA-4 plus anti- PD-1/PD-L1 monoclonal antibodies

Cancer type	Phase	Primary outcome	Dosing regimen	Enrollment number	Status	Results	Reference/ Clinical trials identification number
HIV associated unresectable metastatic solid tumors	Phase 1	MTD of NIVO (time frame: 56 days)	NIVO on D1; study participants in dose level 2 receive IPI on 1st day of every 3rd course of NIVO while those in dose level − 2 receive IPI on 1st day of every 6th course of NIVO; treatment repeated every 14 days for 46 cycles of NIVO	42	Recruiting	NA	NCT02408861
Locally advanced/metastatic solid tumors	Phase 1	Incidence of TRAE (evaluated up to 30 days after completion of therapy) and the incidence of DLT (assessed for 21 days from initiation of treatment); secondary outcomes: OS, PFS, duration of response, OR and best overall response assessed for 3 years	Arm A: atezolizumab + IPI Q3W for 4 cycles; arm B: Interferon alfa-2b (3 doses/week) + atezolizumab Q3W	200	Recruiting	NA	NCT02174172
Advanced incurable solid malignancies	Phase 1b	Determine RP2D tremelimumab with/ without MEDI4736 in patients on treatment with standard of care chemotherapy (assessed up to 2 years)	Tremelimumab (D1 of cycles 1, 3 and 5, or, D1 of cycle 1) with/without MEDI4736 (Q3W)	150	Recruiting	NA	NCT02537418
Advanced solid tumors/relapsed metastatic SCCHN	Phase 1/ Phase 2	Determine the MTD and RP2D; assessment of safety and efficacy of specified treatment regimen; evaluation of ORR for up to 12 months	Experimental part A1: MEDI4736 + AZD9150; A2: MEDI4736 + AZD5069; B1: (patients pre-treated with PD-L1 inhibitor) MEDI4736 + AZD9150; B2: (patients pre-treated with PD-L1 inhibitor) MEDI4736 + AZD5069; B3: (treatment naïve patients) MEDI4736 + AZD9150; B4: (treatment naïve patients) MEDI4736 + AZD5069; B5: AZD9150 until progression, followed by MEDI4736; B6: AZD5069 until progression, followed by MEDI4736; A3: MEDI4736 + AZD5069; A4: MEDI4736 + AZD9150 + tremelimumab; A5: MEDI4736 + AZD5069 + tremelimumab; A6: MEDI4736 + AZD9150; A7: MEDI4736 + AZD5069	147	Recruiting	NA	NCT02499328
Advanced solid tumors	Phase 1	Determine the number of patients with DLT, AE and serious AE; secondary outcomes: OS and ORR, assessed up to 2 years or until death	Arm 1: MEDI4736 Q2W; arm 2: MEDI4736 Q3W; arm 3 (dose expansion): MEDI4736 Q2W; arm 4: MEDI4736 Q4W; arm 5: MEDI4736 + tremelimumab Q4W	264	Recruiting	NA	NCT01938612
Solid tumors	Phase 1/Phase 2	ORR assessed up to 10 years; secondary outcomes: clinical benefit rate assessed for 6 months, PFS and OS evaluated for up to 10 years	IPI on D1 + NIVO on D1, 15 and 29, course to be repeated every 42 days until unacceptable treatment related toxicity or progression of disease	334	Recruiting	NA	NCT02834013 (S1609 trial/DART trial)
Refractory/recurrent solid tumors	Phase 1/Phase 2	RR with IPI + NIVO combination therapy, RR with NIVO, MTD of NIVO, phase 2 dose of IPI + NIVO	IPI + NIVO	352	Recruiting	NA	NCT02304458
Metastatic/advanced solid tumors	Phase 1/Phase 2 RCT	OR rate; secondary outcomes: PFS, OS (time frame: 5 years)	Arm N: NIVO 3 mg/kg Q2W; arm N-I level 1: 4 doses of NIVO 1 mg/kg + IPI 1 mg/kg Q3W, later continued on monotherapy with NIVO 3 mg/kg Q2W; arm N-I level 2: 4 doses of IPI 3 mg/kg + NIVO 1 mg/kg Q3W, later on monotherapy with NIVO 3 mg/kg Q2W; arm N-I level 2b: 4 doses of IPI 1 mg/kg + NIVO 3 mg/kg Q3W, later on monotherapy with NIVO 3 mg/kg Q2W; arm N-I level 2c: IPI 1 mg/kg Q6W + NIVO 3 mg/kg Q3W; arm N-I level 2d: cobimetinib 60 mg/day for 21 days followed by 7 days off + IPI 1 mg/kg Q6W and NIVO 3 mg/kg Q3W	1150	Recruiting, results available for recurrent small cell lung cancer	Recurrent small cell lung cancer: ORR, disease control rate, SD, PR and progressive disease with NIVO (*n* = 40) noted in 18, 38, 20, 18 and 53% patients who received the drug, respectively; NIVO + IPI combination (*n* = 50) exhibited ORR of 17%, SD in 37%, PR in 15%, disease control rate of 54% and progressive disease in 37% patients	NCT01928394 [80]

*Abbreviations: MTD* maximum tolerable dose, *DLT* dose limiting toxicity, *SCCHN* squamous cell carcinoma of the head and neck, *RCT* randomized controlled trial, *NA* not available, *CI* confidence interval, *ORR* overall response rate, *OR* objective response, *OS* overall survival, *PFS* progression free survival, *RR* response rate, *RP2D* recommended phase 2 dose, *TRAE* treatment related adverse events, *NIVO* nivolumab, *IPI* ipilimumab, *PEMBRO* pembrolizumab, *MEDI4736* durvalumab, *OD* once daily dosing, *BID* twice daily, *QID* four times a day; Q(x)W, every (x) weeks, *SD* stable disease, *PR* partial response, D(x), day(x), *AE* adverse events

Recently, the FDA approved the use of pembrolizumab for unresectable/metastatic mismatch repair deficient (dMMR) or microsatellite instability-high (MSI-H) solid tumors and colorectal cancer with progression on prior therapy. This was based upon data from KEYNOTE 012, KEYNOTE 028, KEYNOTE 164, KEYNOTE 016 and KEYNOTE 158 [73–76]. The advised regimen is pembrolizumab 10 mg/kg every 2 weeks or 200 mg every 3 weeks for up to 24 months, unacceptable toxicity or progression of disease. It is of note that this is the first time when a drug has been approved not on the basis of tumor location but a tumor biomarker.

The Dual Anti-CTLA-4 and Anti-PD-1 Blockade in Rare Tumors (DART) trial will evaluate response to combination checkpoint inhibition for a large number of rare tumors in a basket fashion (NCT02834013). In this trial, minimizing toxicity profiles without compromising clinical efficacy is the primary goal. We nominated the low-dose combination therapy with fixed-dose nivolumab and wider interval ipilimumab (nivolumab 240 mg every 2 weeks plus ipilimumab 1 mg/kg every 6 weeks) for assessment in this trial. Our treatment regimen is based on the superior toxicity profile for this regimen observed in CheckMate 012 as compared to the FDA approved combination therapy regimen (nivolumab 1 mg/kg plus ipilimumab 3 mg/kg every 3 weeks for 4 doses followed by monotherapy with nivolumab 3 mg/kg every 2 weeks) in CheckMate 069 [53, 60]. Based on the fact that no significant difference in response was recorded between PD-L1 positive and negative patients receiving combination therapy, we decided to recruit study participants irrespective of tumor PD-L1 status. Through this trial, we expect to provide critical data for expanding the application of low-dose combination therapy in rare tumors.

Recent studies have suggested that sequential administration of immune-checkpoint inhibitors targeting various pathways may benefit cancer patients exhibiting treatment resistance. A multi-center retrospective study evaluated outcomes with ipilimumab and combination therapy with nivolumab and ipilimumab in advanced melanoma patients that previously failed treatment with anti-PD-1 MoAbs [77]. Patients receiving ipilimumab monotherapy were observed to have superior disease control as compared to those receiving combination checkpoint inhibition (42% versus 33%) [77]. Of note, though this was a retrospective study, it was insufficiently powered to detect the difference. A different retrospective analysis of 10 melanoma patients that received ipilimumab after progression on anti-PD-1 therapy found that 1 of 10 patients exhibited partial remission and an additional four patients had stable disease [78]. Similar to the findings of the above and other retrospective studies, the CheckMate 064 documented a higher treatment efficacy in melanoma patients receiving nivolumab prior to ipilimumab versus those receiving ipilimumab prior to nivolumab [57]. However, superior clinical efficacy with the former regimen was associated with an inferior toxicity profile to the latter [57]. Of note, the trial involved a planned switch to reverse sequence at 12 weeks and not at progression. Therefore, it remains unclear if switching to combinatorial regimen at the time of progression is a feasible approach. The outcomes from NCT02731729 trial should perhaps be able to provide some direction on this matter.

## Conclusion

Combination immunotherapy is evolving at a phenomenal pace. In light of initial success in patients with melanoma, efforts to explore the indications for combination checkpoint inhibition with anti-PD-1/PD-L1 and anti-CTLA-4 MoAbs have diversified to a large number of tumor histologies. Several treatment strategies intended for achieving better clinical efficacy and to overcome challenges such as treatment resistance and toxicity associated with the use of immunotherapy agents, are presently under investigation. Of note, the use of low-dose combination checkpoint inhibition with nivolumab and ipilimumab in NSCLC appears to be a promising approach. Alternatively, the use of nivolumab prior to ipilimumab in the induction phase for melanoma patients may be a simple but effective strategy to achieve superior outcomes. Data from ongoing trials is expected to provide vital evidence for validation of the above preliminary findings and facilitate the application of combination checkpoint inhibition on a larger scale.
